# Odd skipped-related 1 controls the pro-regenerative response of fibro-adipogenic progenitors

**DOI:** 10.1038/s41536-023-00291-6

**Published:** 2023-04-05

**Authors:** Georgios Kotsaris, Taimoor H. Qazi, Christian H. Bucher, Hafsa Zahid, Sophie Pöhle-Kronawitter, Vladimir Ugorets, William Jarassier, Stefan Börno, Bernd Timmermann, Claudia Giesecke-Thiel, Aris N. Economides, Fabien Le Grand, Pedro Vallecillo-García, Petra Knaus, Sven Geissler, Sigmar Stricker

**Affiliations:** 1 Institute of Chemistry and Biochemistry, Musculoskeletal Development and Regeneration Group, Freie Universität Berlin, Thielallee 63, 14195 Berlin, Germany; 2 Berlin-Brandenburg School for Regenerative Therapies, Charité - Universitätsmedizin Berlin, 13353 Berlin, Germany; 3 Berlin Institute of Health at Charité - Universitätsmedizin Berlin, BIH Julius Wolff Institute, Augustenburger Platz 1, 13353 Berlin, Germany; 4 Department of Bioengineering, University of Pennsylvania, 19104 Philadelphia, USA; 5 Berlin Institute of Health at Charité - Universitätsmedizin Berlin, BIH Center for Regenerative Therapies (BCRT), Charitéplatz 1, 10117 Berlin, Germany; 6 International Max Planck Research School for Biology and Computing IMPRS-BAC, Berlin, Germany; 7 Max Planck Institute for Molecular Genetics, Ihnestrasse 73, 14195 Berlin, Germany; 8 Institute of Chemistry and Biochemistry, Cell Signaling Group, Freie Universität Berlin, Thielallee 63, 14195 Berlin, Germany; 9 Institut NeuroMyoGène, CNRS UMR 5261, Inserm U1315, Université Claude Bernard Lyon 1, 69008 Lyon, France; 10 Regeneron Pharmaceuticals Inc., Tarrytown, NY USA; 11 Berlin Center for Advanced Therapies (BECAT), Charité Universitätsmedizin Berlin, Augustenburger Platz 1, Berlin, Germany; 12 Present Address: Weldon School of Biomedical Engineering, Purdue University, 47907 West Lafayette, IN USA

**Keywords:** Regeneration, Muscle stem cells, Mesenchymal stem cells

## Abstract

Skeletal muscle regeneration requires the coordinated interplay of diverse tissue-resident- and infiltrating cells. Fibro-adipogenic progenitors (FAPs) are an interstitial cell population that provides a beneficial microenvironment for muscle stem cells (MuSCs) during muscle regeneration. Here we show that the transcription factor Osr1 is essential for FAPs to communicate with MuSCs and infiltrating macrophages, thus coordinating muscle regeneration. Conditional inactivation of Osr1 impaired muscle regeneration with reduced myofiber growth and formation of excessive fibrotic tissue with reduced stiffness. Osr1-deficient FAPs acquired a fibrogenic identity with altered matrix secretion and cytokine expression resulting in impaired MuSC viability, expansion and differentiation. Immune cell profiling suggested a novel role for Osr1-FAPs in macrophage polarization. In vitro analysis suggested that increased TGFβ signaling and altered matrix deposition by Osr1-deficient FAPs actively suppressed regenerative myogenesis. In conclusion, we show that Osr1 is central to FAP function orchestrating key regenerative events such as inflammation, matrix secretion and myogenesis.

## Introduction

Skeletal muscle, which accounts for 30–40% of the total body mass in mammals, is one of the few tissues capable of scarless healing. However, large-scale trauma or muscle disease often lead to replacement of damaged tissue by fibrous and fatty infiltrates^1^. The regenerative capacity of skeletal muscle depends on tissue-resident muscle stem cells (MuSCs) that are essential for skeletal muscle regeneration^2,3^. MuSCs are rapidly activated, and upon tissue damage start to proliferate and give rise to a progenitor pool capable of replacing damaged muscle fibers^4,5^. During regeneration, myogenesis is tightly connected to an interplay of multiple other cell types. Skeletal muscle regeneration follows the general principle of wound healing and begins with an initial inflammatory response characterized by infiltration of immune cells, which represent the first wave of cells expanding in the injured area^6^. Among immune cells, macrophages play a key role in orchestrating the regeneration process. Classically activated M1-like pro-inflammatory macrophages are required for the clearance of tissue debris, attraction and modulation of further immune cells and activation of MuSCs^7^. Subsequently, these macrophages convert into alternatively activated M2-like restorative phenotypes, which promotes muscle progenitor differentiation into myocytes and their fusion to new myofibers^4,5,8,9^. Immune cell infiltration in the injury area is also associated with the immediate activation and expansion of tissue-resident stromal cells called fibro-adipogenic progenitors (FAPs)^10^. FAPs, originally identified by expression of the cell surface markers Sca-1 or PDGFRα^11,12^, generate a beneficial microenvironment for regeneration in part via secreted signaling molecules and via the formation of a transient extracellular matrix (ECM)^11,13–15^. While FAPs are essential for effective muscle regeneration under physiological conditions^3,16^, they are the source of fibrosis and fatty infiltration under degenerating or chronic inflammatory states^14,17–21^. To control transitional FAP pool expansion and prevent fibro-fatty infiltration, classically activated M1-like macrophages induce FAP apoptosis via TNFα in mid-regeneration thus limiting transient ECM production and making way for regenerating muscle fibers^22^. Conversely, alternatively activated M2-like macrophages create a TGFβ-rich environment that induces differentiation of FAPs into myofibroblasts, which increasingly synthesize new ECM components^15,22^. Accordingly, many myopathies, including amyotrophic lateral sclerosis and Duchenne muscular dystrophy, are associated with exacerbated inflammatory responses, resulting in deregulated FAP function, excessive fibrotic tissue formation, and loss of muscle function^21,23^.

Despite increasing knowledge about the role of FAPs during the regenerative process, no intrinsic transcriptional regulator is known that controls key aspects of their different functions. We previously identified the zinc finger transcription factor Odd skipped-related 1 (Osr1) as a key regulator of the pro-myogenic function in an embryonic FAP-like cell population, which also is a developmental source of adult FAPs^24^. While *Osr1* reporter gene and protein expression was undetectable in homeostatic muscle, it was reactivated during muscle regeneration^25^. Here we show that *Osr1* is required for FAP function during skeletal muscle regeneration. Loss of *Osr1* leads to a pro-fibrotic orientation of FAPs and impairs both FAP-macrophage and FAPs-MuSC communication networks resulting in impaired regenerative myogenesis and persistent fibrosis. This demonstrates that Osr1 is a key transcriptional regulator of FAP regenerative function protecting FAPs from assuming a detrimental pro-fibrotic and anti-myogenic state.

## Results

### Conditional inactivation of Osr1 impairs muscle regeneration

**Fig. 1:**
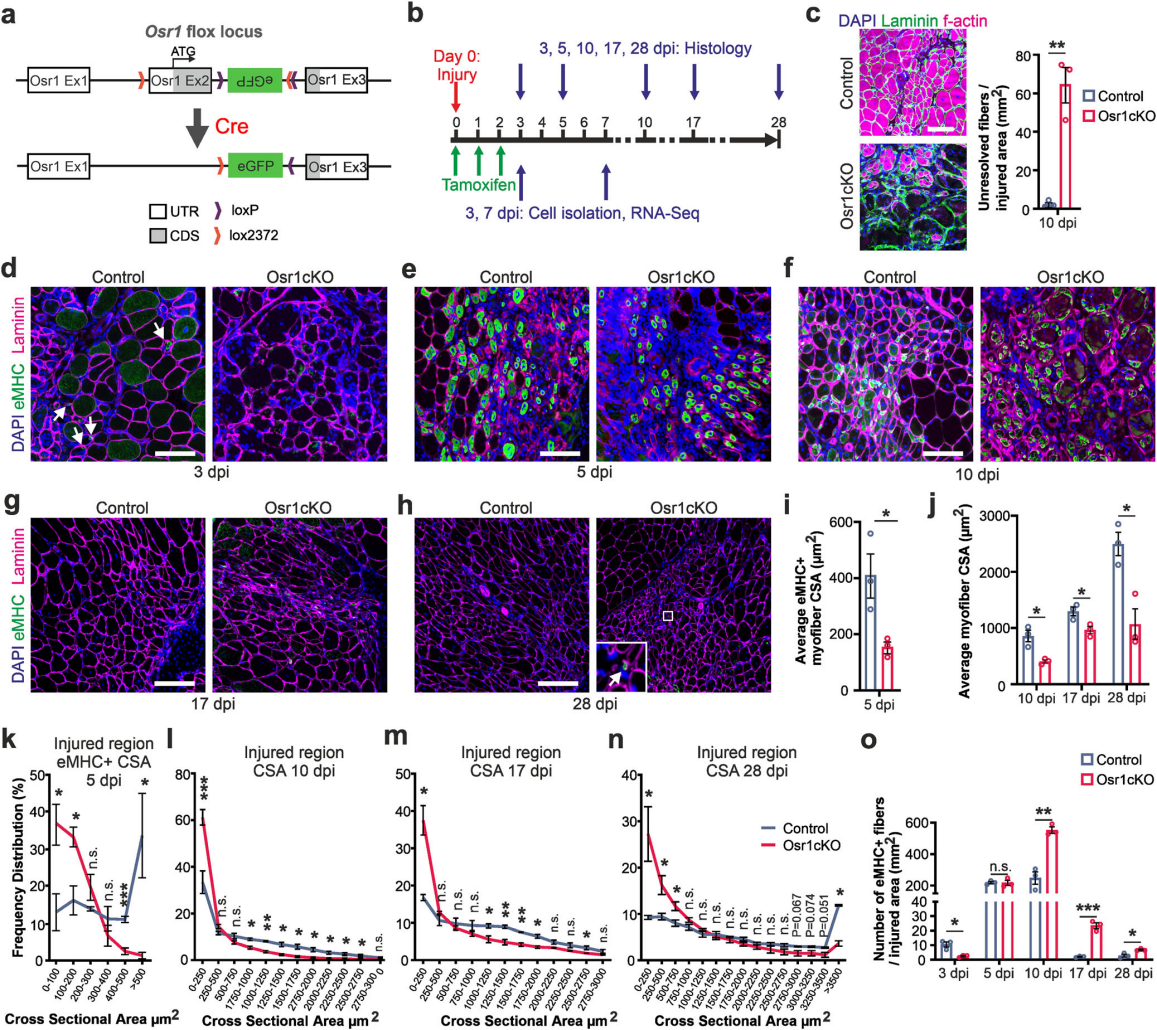
Conditional inactivation of Osr1 impairs muscle regeneration. **a** Schematic representation of the *Osr1* conditional allele and Cre-mediated locus recombination **b** Schematic depiction of Tamoxifen administration and analysis time points (dpi: days post-injury). **c** Phalloidin labeling for f-actin at 10 dpi to detect unresolved ghost fibers; quantification shown right (*n* = 3). **d**–**h** Immunolabeling for embryonal myosin heavy chain (eMHC) and Laminin on control and Osr1cKO muscle sections at indicated dpi; nuclei were stained for DAPI. **i** Quantification of eMHC+ myofiber cross sectional area (CSA) in the injury region of control and Osr1cKO muscle at 5 dpi (*n* = 3). **j** Quantification of myofiber cross sectional area (CSA) in the injury region of control and Osr1cKO muscle at 10, 17 and 28 dpi (*n* = 3). **k** eMHC+ Myofiber size frequency distribution in the injury region of control and Osr1cKO muscle at 5 dpi (*n* = 3). **l**–**n** Myofiber size frequency distribution in the injury region of control and Osr1cKO muscle at 10, 17 and 28 dpi (*n* = 3). **o** Quantification of eMHC positive fibers per area at indicated dpi (*n* = 3). Data are represented as mean ± SEM; *P*‐value calculated by two‐sided unpaired *t* test between control and Osr1cKO samples for each time point; *p < 0.05, **p < 0.01, ***p < 0.001. *N*‐numbers indicate biological replicates (mice per genotype). Scale bars: 100 μm in (**c**–**h**).

First, *Osr1* expression was analyzed in the single cell dataset from Oprescu et al.^26^, confirming *Osr1* expression in FAPs throughout muscle regeneration (Supplementary Fig. 1a) in agreement with our previous data^25^. Osr1-flox mice generated from an *Osr1* multifunctional allele^25^ were mated to CAGG-CreER animals to allow timed inactivation of *Osr1* based on tamoxifen delivery. Cre-mediated recombination inactivates Osr1 by replacing it with eGFP, which can be used to track recombined cells (Fig. 1a). Tamoxifen was administered concomitant to freeze-pierce injury of the tibialis anterior muscle (TA), and on the following two days (Fig. 1b). Expression of GFP from the recombined *Osr1* allele was analyzed via flow cytometry and found exclusively in Lin- (non-hematopoietic, non-endothelial) cells (Supplementary Fig. 1b). To further confirm specific *Osr1* expression in FAPs, FACS-isolated FAPs (Lin-/Sca1+), MuSCs (Lin-/α7-integrin+) and Lin+ (CD31, CD45, TER-119) cells were analyzed by RT-qPCR. *Osr1* was only detected in FAPs, not in MuSCs or Lin+ cells (Supplementary Fig. 1c). Osr1-deficient Osr1^flox/flox^;CAGG^CreER^ animals are further termed Osr1cKO. *Osr1* mRNA expression was efficiently decreased by 80% in FAPs (Supplementary Fig. 1c) or total muscle samples (Supplementary Fig. 1d) of 3 dpi Osr1cKO mice.

No major histological differences were observed between Osr1cKO and control muscles at 3 and 5 dpi (Supplementary Fig. 2). Both genotypes showed hallmarks of degeneration of injured muscle tissue driven by the initial pro-inflammatory phase. At 10 dpi histology indicated accumulation of granulation tissue and abundance of degenerating fibers in Osr1cKO muscle compared to controls (Supplementary Fig. 2). Indeed, in Osr1cKO mice filamentous actin (f-actin)-negative degenerating “ghost” fibers persisted at 10dpi (Fig. 1c), whereas degenerating tissue was already completely resolved in the controls.

Overall, regenerating myofibers appeared smaller and more variable in size in Osr1cKO muscle (Supplementary Fig. 2). Immunolabeling for laminin to outline muscle fibers and for embryonal myosin heavy chain (eMHC) to label newly regenerating fibers confirmed impaired post-injury myofiber growth in Osr1cKO animals (Fig. 1d–h). The average size (cross sectional area, CSA) of eMHC+ myofibers at 5 dpi was reduced in Osr1cKO muscle to less than 50% of control levels (Fig. 1i). At 10, 17 and 28 dpi myofibers in the injury area of Osr1cKO mice were 30–60% smaller compared to corresponding controls (Fig. 1j). While a steep increase in fiber size was observed in the controls especially between 17 and 28 dpi, fiber size remained constant in the Osr1cKO. Plotting discrete size windows (CSA distribution frequency) confirmed a shift towards extremely small fiber calibers in Osr1cKO muscle, with approx. 70% of eMHC+ fibers having a CSA of less than 200 µm^2^ at 5 dpi (Fig. 1k), and approx. 50% of fibers having a CSA less than 500 µm^2^ at 10, 17 and 28 dpi (Fig. 1l–n).

Quantification of actively regenerating eMHC+ fibers within the injury area showed a delay in formation and maturation of new fibers. Compared to the controls, Osr1cKO muscles exhibited a significantly lower amount of eMHC+ fibers at 3 dpi (Fig. 1d, o), which was equalized at 5 dpi (Fig. 1e, o). Conversely, a higher proportion of eMHC+ fibers was observed in Osr1cKO muscle at 10 dpi (Fig. 1f, o), which accumulated around apparently non-resolving ghost fibers (Fig. 1f). At 17 and 28 dpi eMHC+ fibers had almost completely disappeared in controls but persisted in the Osr1cKO (Fig. 1g, h, o).

In conclusion, inactivation of Osr1 leads to impaired degenerating tissue resolution and delayed formation, maturation and growth of regenerating myofibers.

### Loss of Osr1 leads to reduced regenerating tissue stiffness and persistent fibrosis

**Fig. 2:**
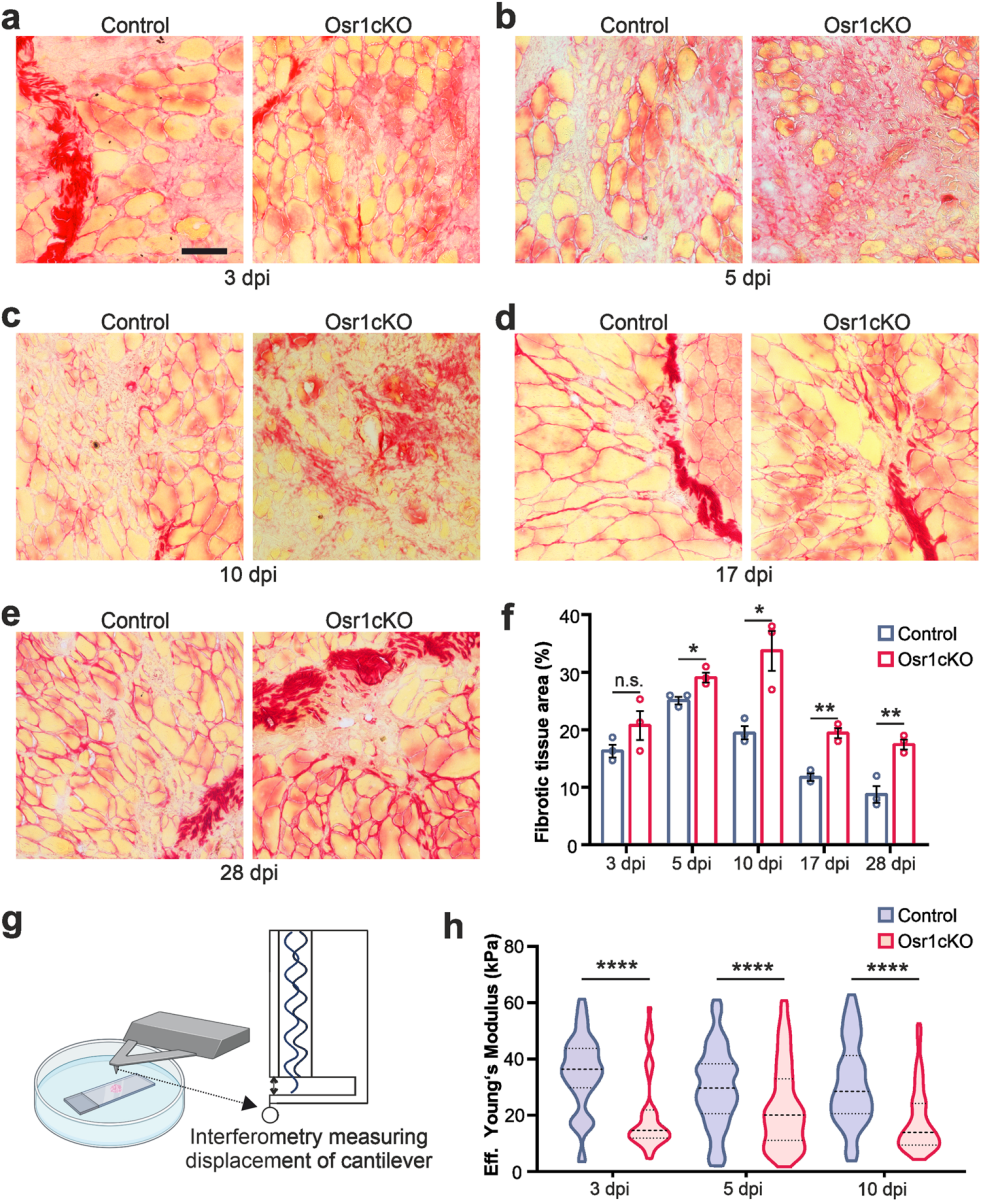
Loss of Osr1 results in persistent fibrosis and tissue softening. **a**–**e** Picrosirius red staining on control and Osr1cKO muscle sections at indicated days post-injury (dpi). **f** Quantification of picrosirius red staining (*n* = 3). Data are presented as mean ± SEM. **g** Schematic representation of nanoindentation method to assess tissue stiffness. **h** Quantification of nanoindentation stiffness measurements of the injured area in muscle tissue sections of control and Osr1cKO mice (*n* = 3). Violin plots show full data range, mean value, first and third quartiles are indicated. *P*‐value in (**f**, **h**) calculated by two‐sided unpaired *t* test between control and Osr1cKO samples for each time point; *p < 0.05, **p < 0.01, ****p < 0.0001. *N*‐numbers indicate biological replicates (mice per genotype). Scale bars: 100 μm in (**a**–**e**).

Immunolabeling for laminin (Fig. 1d–h) and collagen type VI (Supplementary Fig. 3a) indicated an increase in ECM in Osr1cKO muscle. Picrosirius red staining was performed to evaluate ECM deposition. No difference was observed at 3 dpi between control and Osr1cKO muscle, indicating the initial transient fibrotic response as a normal feature of the regeneration process (Fig. 2a, f). Starting at 5 dpi, a significant increase in ECM deposition was found in Osr1cKO muscle (Fig. 2b, f). While ECM remodeling in controls led to a marked decrease in picrosirius red staining between 5 and 10 dpi, it peaked at 10 dpi in Osr1cKO animals, and started to decrease between 10 and 17 dpi (Fig. 2c, d, f). At 17 and 28 dpi, Osr1cKO animals still showed approx. twice as much picrosirius red staining than controls (Fig. 2d–f) indicating persistent fibrosis.

Fibrotic scarring is typically associated with increased tissue stiffness^27^, as it is also observed in *mdx* animals used as a model for Duchenne muscular dystrophy^28^. However, these observations are typically made by assessing passive tissue stiffness at a late time point of disease^29,30^. To assess the impact of altered tissue remodeling on the mechanical properties of the regenerating tissue during the initial phase of transient pro-regenerative ECM formation, nanoindentation was performed (Fig. 2g). Dynamic alterations in tissue stiffness were first defined in wild type mice by comparing the injured region of damaged muscles at 3, 4 and 5 dpi with the corresponding intact (contralateral) muscle. This showed a transient stiffness increase at 3 and 4 dpi, which decreased back to the range of uninjured levels already at 5 dpi (Supplementary Fig. 3b). In regenerating Osr1cKO muscles no transient increase in stiffness was observed, but rather a tissue softening occurred at 3 dpi (Fig. 2h). Injured Osr1cKO muscle consistently exhibited lower stiffness than controls at 5 and even at 10 dpi (Fig. 2h). Taken together, loss of Osr1 resulted in a softened transient ECM during regeneration followed by persistent fibrosis.

### Osr1-deficient FAPs have an intrinsic defect in pool expansion

**Fig. 3:**
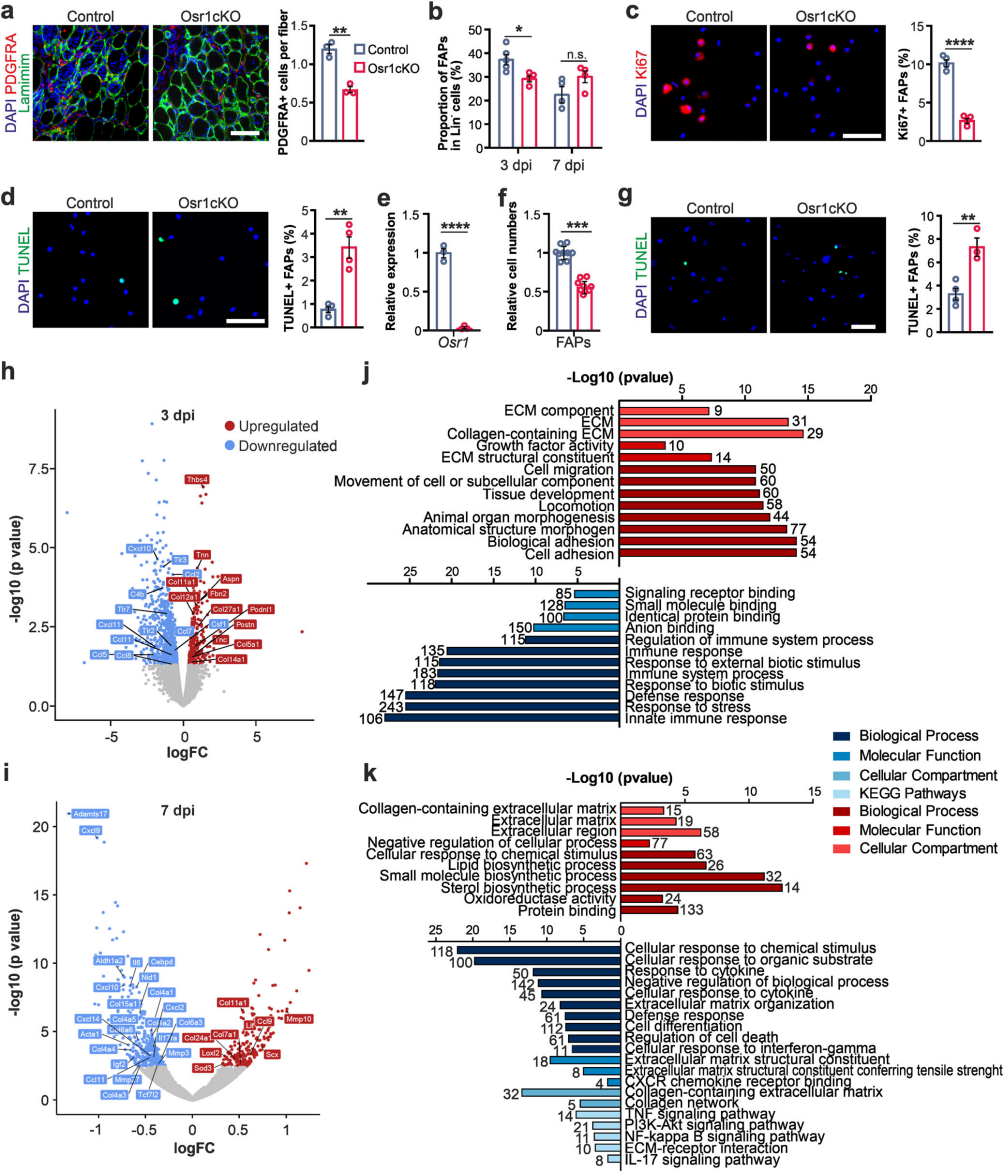
Osr1 deletion affects FAP expansion and transcriptomic response after acute injury. **a** PDGFRA staining on 3 dpi tissue sections of control and Osr1cKO TA muscle; myofibers are outlined by Laminin, nuclei are stained with DAPI. Quantification of PDGFRA+ cells per myofiber in the regenerative area is shown right (*n* = 3 animals per genotype). **b** Flow cytometry analysis of FAP numbers at 3 and at 7 dpi (at 3 dpi *n* = 5 for control and *n* = 4 for Osr1cKO, at 7 dpi *n* = 4 each). **c** Ki67 labeling of 7 dpi FAPs isolated by FACS; quantification right (*n* = 4). **d** TUNEL labeling 7 dpi FAPs isolated by FACS; quantification right (*n* = 4). **e** RT-qPCR analysis of Osr1 expression after 4OHT-mediated in vitro recombination of FAPs isolated from control and Osr1cKO animals. **f** Cell numbers of in vitro recombined FAPs after 6 days of culture in growth medium. **g** TUNEL labeling on in vitro recombined FAPs after 4 days of culture in growth medium. **h**, **i** Volcano plot of DE genes between Osr1^flox/+^;CAGG-Cre^+^ (control) and Osr1cKO FAPs at 3 or 7 dpi (*n* = 2). **j**, **k** GO analysis of genes upregulated (red) or downregulated (blue) in Osr1cKO FAPs at 3 or 7 dpi (*n* = 2). Data are presented as mean ± SEM; *P*‐value calculated by two‐sided unpaired t‐test between control and Osr1cKO samples for each time point; *p < 0.05, **p < 0.01, ***p < 0.001, ****p < 0.0001. *N*‐numbers indicate biological replicates (mice per genotype). Scale bars: 100 μm in (**a**, **g**); 50 μm in (**c**, **d**).

We next assessed possible direct effects of Osr1 depletion on FAPs. Quantification of PDGFRA + FAPs on tissue sections at 3 dpi showed a decreased number of FAPs in the regenerating region of Osr1cKO animals compared to the control (Fig. 3a). This was confirmed by flow cytometry analysis at 3 dpi (Fig. 3b), at 7 dpi, however, FAP numbers were not significantly altered (Fig. 3b). FAPs isolated by fluorescence-activated cell sorting (FACS) and immediately stained after isolation showed approx. 80% decreased Ki67 labeling (Fig. 3c), and approx. three-fold increased apoptosis rate (Fig. 3d).

To assess whether these defects were cell autonomous, we performed in vitro recombination of the Osr1^flox/flox^ allele in Osr1cKO FAPs. FAPs were isolated by pre-plating, yielding cells phenotypically and biochemically similar to FAPs isolated by FACS^31^. 4-hydroxytamoxifen (4OHT) treatment resulted in an approx. 98% decrease of *Osr1* mRNA expression (Fig. 3e). In line with in vivo results, also the in vitro recombined FAPs showed significantly reduced cell numbers after 6 days of culture in growth medium (Fig. 3f) and increased apoptosis (Fig. 3g) suggesting that Osr1 is required for FAP viability.

### Osr1-deficient FAPs show altered cytokine and ECM gene expression profiles

To gain deeper insight into intrinsic effects of Osr1 depletion, we performed transcriptome analysis of FAPs isolated by FACS at 3 and 7 dpi. To enrich FAPs from the injury region, cells with active *Osr1* expression were isolated based on eGFP expression from the recombined Osr1^flox^ allele (Supplementary Fig. 1b). Efficient deletion of floxed exon 2 and consequent lack of exon 3 expression was confirmed in the RNA Sequencing (RNA-Seq) data (Supplementary Fig. 4a). RNA-Seq analysis revealed a total of 950 differentially expressed genes (DEG), of which 261 were upregulated and 689 were downregulated in homozygous Osr1cKO FAPs compared with heterozygous controls at 3 dpi (Fig. 3h). At 7 dpi, 544 DEGs were determined, of which 206 were up- and 338 were downregulated in Osr1cKO FAPs (Fig. 3i). We then performed gene ontology (GO) annotation clustering of the DEGs according to cellular component, biological process and signaling pathway. This revealed that a significant number of DEGs upregulated in Osr1cKO FAPs at 3 dpi were associated with the ECM (GO:0031012), “collagen-containing ECM” (GO: 0062023), “extracellular structural constituent” (GO:0005201) or cell-matrix interaction such as “cell adhesion” (GO:0007155) (Fig. 3j). Upregulated DEGs at 7 dpi were also mainly associated with terms related to the ECM (Fig. 3k). DEGs downregulated in Osr1cKO FAPs showed enrichment of genes related to the “innate immune response” (GO:0045087) or “regulation of immune system” (GO:0002682) at 3 dpi (Fig. 3j) and enrichment of genes belonging to “response to cytokine” (GO:0034097) and “defense response” (GO:0034097) at 7 dpi (Fig. 3k). Accordingly, analysis of genes commonly deregulated at both time points (total: 96 genes: 32 up- and 45 downregulated at both time points, 19 genes with opposite regulation; Supplementary Fig. 4b) confirmed that they were associated with GO terms related to “collagen-containing extracellular matrix” and “defense response,” respectively. (Supplementary Fig. 4c, d).

In summary, loss of Osr1 impairs initial FAP expansion and leads to a sustained shift in their transcriptional profile. These transcriptional alterations might affect several key functions attributed to FAPs during regeneration^6^, including the synthesis and remodeling of the transient ECM, but also their immunomodulatory properties.

### Loss of Osr1 disrupts FAP-immune cell interplay and prevents regenerative macrophage polarization

**Fig. 4:**
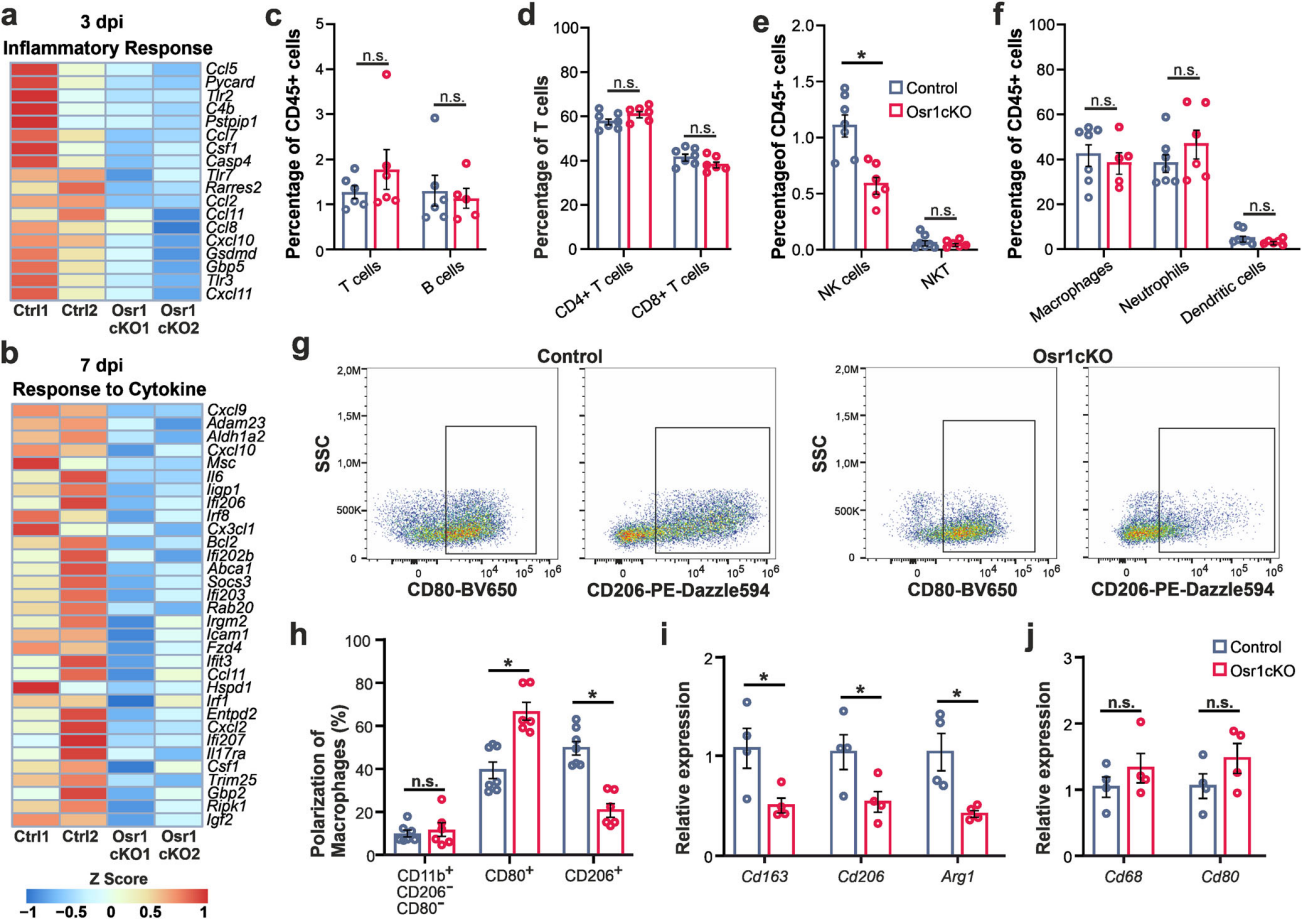
Loss of Osr1 leads to altered macrophage polarization during skeletal muscle regeneration. **a**, **b** Heat maps of DE genes in 3 or 7 dpi control vs. Osr1cKO FAPs belonging to GO terms “Inflammatory response” and “Response to cytokine” (*n* = 2). **c**–**f** Relative quantification by flow cytometry of overall T- and B-cells (c), CD4 and CD8 T cells (**d**), NK/NKT cells (**e**) and myeloid subpopulations (f) isolated from control or Osr1cKO 3 dpi muscle (*n* = 7 for control and *n* = 6 for Osr1cKO). **g** Representative FACS plots of CD80 / CD206 macrophage polarization analysis. **h** Quantification of non-activated (CD11b + /CD206−/CD80−) and classically activated M1-like (CD80+) or alternatively activated M2-like (CD206+) macrophages in control or Osr1cKO muscle at 3 dpi. **i**, **j** RT-qPCR analysis of *Cd163*, *Cd206* and *Arg1*, or *Cd68* and *Cd80* mRNA in whole muscle lysate (*n* = 4). Data are represented as mean ± SEM; *P*‐value calculated by Mann–Whitney test in (**c**–**f**) and by two‐sided unpaired *t* test in (**h**–**j**) between control and Osr1cKO samples for each cell type; *p < 0.05. *N*‐numbers indicate biological replicates (mice per genotype).

Since the transcriptome analysis indicated altered immunomodulatory properties of Osr1cKO FAPs, we investigated possible implications for the interplay between FAPs and immune cells during muscle regeneration. Heatmap depiction showed a global downregulation of genes including numerous secreted signaling molecules and cytokines, belonging to GO groups “inflammatory response” and “response to cytokine”, in Osr1cKO FAPs (Fig. 4a, b).

Flow cytometry analysis of 3 dpi muscles confirmed the presence of major immune cell subpopulations in Osr1cKO and control animals (Supplementary Fig. 5a). Equal total live cell numbers and, within live cells, equal fractions of CD45+ cells (leukocytes) were isolated from control or Osr1cKO muscle (Supplementary Fig. 5b). Numbers of B- and T-cells were globally unaltered in 3 dpi Osr1cKO muscle (Fig. 4c, Supplementary Fig. 5c), as were T-cell-subsets (Fig. 4d). Low levels of circulating memory T-cells in the blood confirm that animals used are immunocompetent but still naïve in their immunological memory (Supplementary Fig. 5d). Blood samples also showed no differences in the systemic levels of monocytes or neutrophils between controls and Osr1cKO at 3 dpi (Supplementary Fig. 5e) excluding systemic effects. Significantly reduced levels of natural killer (NK) cells, but not NKT cells, were found in 3 dpi Osr1cKO muscles (Fig. 4e). No significant differences in the relative overall numbers of macrophages, neutrophils or dendritic cells (Fig. 4f, Supplementary Fig. 5c), or dendritic cell subsets (Supplementary Fig. 5f) were detected between Osr1cKO and control 3 dpi muscles. This together argues against a global effect of Osr1 depletion in FAPs on immune cell infiltration.

As controlled macrophage polarization is essential for efficient muscle regeneration^8,9^, we analyzed classically activated M1-like (CD80+), alternatively activated M2-like (CD206+) and non-activated (CD11b+/CD80-/CD206-) macrophage subsets. This revealed significantly increased relative numbers of M1-like CD80 + macrophages in Osr1cKO muscles, which was accompanied by lower levels of M2-like CD206 + macrophages (Fig. 4g, h). This was confirmed on tissue sections (Supplementary Fig. 5g). While control muscles had a ratio of approximately 1:1 between both macrophage subsets, this ratio was shifted to 3:1 in Osr1cKO muscle (Fig. 4h). Note that the levels of non-activated macrophages remained unaltered (Fig. 4h) suggesting unchanged overall macrophage activation but perturbed polarization in injured Osr1cKO muscles. This finding was confirmed by qPCR examination of whole muscle lysate, which revealed significantly lower expression of *Cd163*, *Cd206* and *Arg1* (alternatively activated M2-like macrophage markers) in Osr1cKO muscles (Fig. 4i). Expression of *Cd68* and *Cd80* (classically activated M1-like macrophage markers) was not significantly changed, but tended to be increased in Osr1cKO muscle (Fig. 4j). Classically activated M1-like macrophages limit the expansion of FAPs through TNFα-induced apoptosis^22^. However, a targeted analysis of transcriptome data showed that genes related to the TNFα signaling pathway are largely downregulated in Osr1cKO FAPs (Supplementary Fig. 6), suggesting that the cells may be less responsive. In summary, Osr1 depletion does not impair the timing of immune cell infiltration into the injured muscle, but leads to altered macrophage polarization.

### Loss of Osr1 affects MuSCs during regeneration

**Fig. 5:**
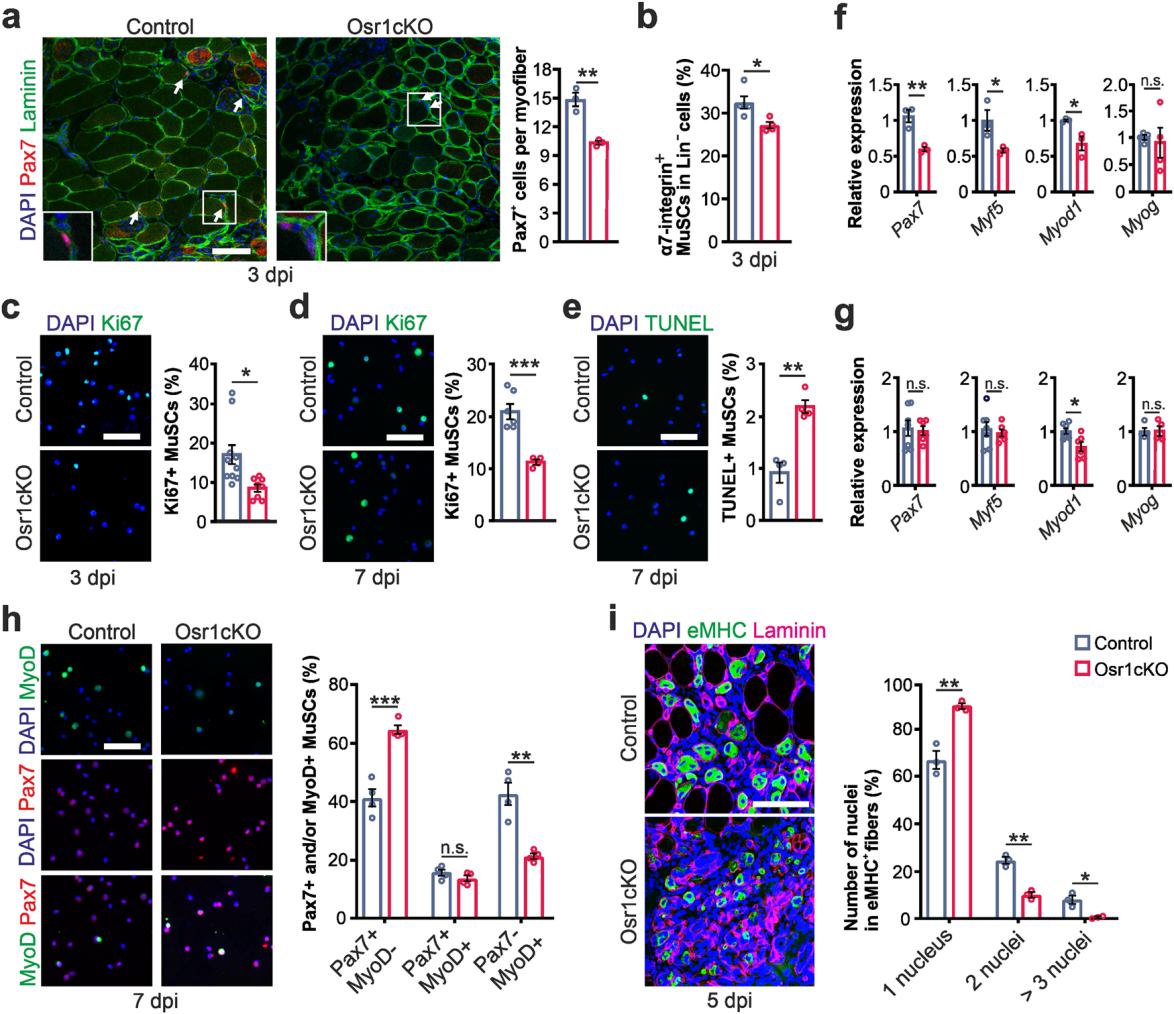
Non-cell autonomous defect of MuSCs in Osr1cKO muscle. **a** Pax7 staining on 3 dpi tissue sections of control and Osr1cKO TA muscle; myofibers are outlined by Laminin, nuclei are stained with DAPI. Quantification of Pax7+ cells per myofiber in the regenerative area is shown right (*n* = 3 animals per genotype). **b** Flow cytometric analysis of MuSC numbers in Lin- cells at 3 dpi (*n* = 5 for control and *n* = 4 for Osr1cKO). **c**, **d** Ki67 labeling of cytospun MuSCs freshly isolated by FACS at 3 and 7 dpi; representative images left, quantification right (at 3 dpi *n* = 10 for control and *n* = 8 for Osr1cKO, at 7 dpi *n* = 6 for control and *n* = 4 for OsrcKO). **e** TUNEL labeling of cytospun MuSCs freshly isolated by FACs at 7 dpi; representative images left, quantification right (*n* = 4). **f**, **g** RT-qPCR for *Pax7*, *Myf5, Myod1* and *Myog* performed on MuSCs isolated by FACs at 3 dpi and 7 dpi (at 3 dpi *n* = 3, at 7 dpi *n* = 7 for control and *n* = 6 for Osr1cKO; each dot represents the mean of three technical replicates from one biological replicate). **h** Immunolabeling for Pax7 and MyoD on cytospun MuSCs freshly isolated by FACs at 7 dpi; quantification shown right (*n* = 4). **i** Immunolabeling for eMHC, Laminin on 5 dpi tissue sections of control and Osr1cKO TA muscle; nuclei are stained with DAPI. Quantification of myonuclei per eMHC+ fiber is shown right. Data are presented as mean ± SEM; *P*‐value calculated by two‐sided unpaired *t* test; *p < 0.05, **p < 0.01, ***p < 0.001. *N*‐numbers indicate biological replicates (mice per genotype). Scale bars: 100 μm in (**a**, **i**); 50 μm in (**c**–**e**, **h**).

We next investigated whether the observed delay of muscle regeneration was preceded by altered MuSC function. Immunolabeling for Pax7 showed an approx. 30% decrease in MuSC numbers in the injury area of Osr1cKO muscle at 3 dpi (Fig. 5a). Despite the small lesion size in our model, flow cytometry analysis still detected an approx. 5% reduction of MuSCs levels in Osr1cKO TA muscles at 3 dpi compared to controls (Fig. 5b). Ki67 immunostaining of freshly FACS-isolated MuSCs at 3 and 7 dpi showed an approx. 60% reduction of Ki67+ MuSCs in Osr1cKO animals (Fig. 5c, d). TUNEL staining indicated increased apoptosis of MuSCs in Osr1cKO mice (Fig. 5e).

To gain insight into the activation state of MuSCs at 3 and 7 dpi, mRNA expression of *Pax7*, *Myf5*, *Myod1* and *Myogenin* (*Myog*) was quantified in FACS-sorted MuSCs (Fig. 5f, g). The expression of *Pax7*, *Myf5* and *Myod1* was significantly reduced in MuSCs of Osr1cKO mice at 3 dpi (Fig. 5f), indicating a defect in the resting and activated states of MuSCs. While *Myf5* and *Pax7* levels were comparable to controls at 7dpi, expression of *Myod1* remained significantly lower at 7 dpi in MuSCs from Osr1cKO mice (Fig. 5g). Expression of *Myog* did not change at both days, arguing against precocious differentiation. Immunostaining for Pax7 and MyoD on freshly FACS-isolated MuSCs demonstrated a 30% relative increase in Pax7 + /MyoD− self renewing cells in the Osr1cKO animals, a similar number of Pax7 + /MyoD+ transit amplifying cells, and a 40% reduction in Pax7-/MyoD+ committed cells (Fig. 5h). We further used quantification of nuclei numbers in eMHC+ fibers at 5 dpi as a proxy for myoblast fusion. This demonstrated a reduced myonuclear accretion in Osr1cKO animals (Fig. 5i) in line with impaired myoblast differentiation. These results indicate that loss of Osr1 expression in FAPs affects the function of MuSCs in a non-cell autonomous manner and compromises their activation, proliferation, differentiation and survival, contributing to the delayed muscle regeneration.

### Osr1cKO FAPs affect myogenesis via TGFβ signaling

**Fig. 6:**
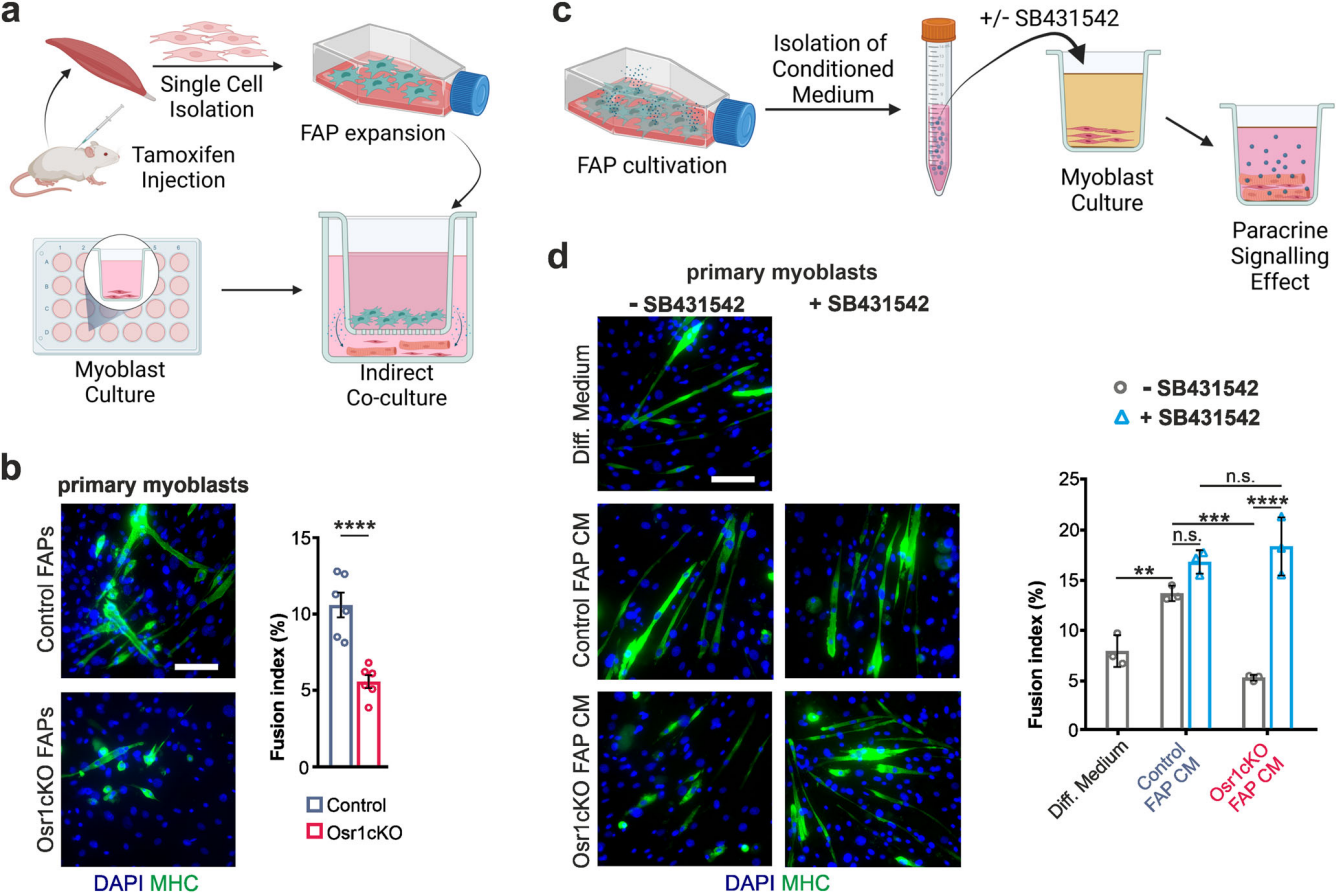
Osr1cKO FAPs inhibit myogenesis via TGFβ signaling. **a** Schematic representation of the transwell assay. **b** Immunolabeling for MHC to detect myotube formation from primary myoblasts co-cultured with control or Osr1cKO FAPs; quantification of fusion index is shown right (*n* = 6). **c** Schematic representation of conditioned medium (CM) assay. **d** Immunolabeling for MHC to detect myotube formation from primary myoblasts in differentiation medium or differentiation medium supplemented with control or Osr1cKO CM, with or without TGFβ pathway inhibitor SB431542; quantification of fusion index is shown right (*n* = 3). Data are mean ± SEM; *P*‐value calculated by two‐sided unpaired *t* test in (**b**) and ANOVA in (**d**); **p < 0.01, ***p < 0.001, ****p < 0.0001. *N*‐numbers indicate biological replicates (mice per genotype). Scale bars: 100 μm in (**b**, **d**).

We next aimed to unravel how Osr1-deficient FAPs may affect MuSC function. FAPs were proposed to promote myogenesis via secreted factors^6,32^. To test for paracrine effects of Osr1cKO FAPs on myogenesis, we performed indirect transwell co-culture experiments (Fig. 6a). We used 7 dpi Osr1cKO or control FAPs that were isolated from contralateral muscles of injured animals to achieve an activated “alert” state^33^, and primary myoblasts from wild type mice or C2C12 cells. Effective recombination of the *Osr1* locus in isolated FAPs was corroborated by RT-qPCR (Supplementary Fig. 7a). Four days after induction of differentiation, fusion of primary myoblasts into myofibers was approx. 50% decreased and fusion of C2C12 cells was almost 33% decreased in co-cultures with Osr1cKO FAPs compared control FAPs (Fig. 6b, Supplementary Fig. 7b). Since Osr1cKO FAPs show reduced proliferation, we tested for FAP numbers in the transwell inserts on the day of analysis; equal numbers in both conditions (Supplementary Fig. 7c) excluded altered FAP numbers as a confounder.

To confirm this result, we used conditioned medium (CM) generated from Osr1cKO or control FAPs (Fig. 6c). Myogenesis of C2C12 cells was reduced by approx. 80% in cultures with CM from Osr1cKO FAPs compared to control media (Supplementary Fig. 7d). These results suggest that Osr1cKO FAPs actively suppress myogenesis via secreted factors. Subsequent search of our transcriptome data for signaling molecules known to negatively affect myogenesis revealed TGFβ pathway-associated GO terms enriched in Osr1cKO FAPs (Supplementary Fig. 6a, b), and *Tgfb* genes were upregulated (Supplementary Fig. 7e) in Osr1cKO FAPs. Upregulation of *Tgfb1* was also confirmed in FAPs after in vitro recombination (Supplementary Fig. 7f), suggesting that increased *Tgfb1* expression is independent from the injury microenvironment.

TGFβ signaling inhibits myogenic differentiation and myoblast fusion by suppressing *Myod1* and *Myog* expression^34,35^ and the control of actin cytoskeleton-related genes^36^. To assess the relevance of TGFβ signaling, TGFβ Type1 receptor kinase inhibitor SB431542 was used. Treatment of C2C12 cells alone with SB431542 reduced phosphorylated SMAD 2/3 levels (Supplementary Fig. 8a), resulted in elevated myogenin and MHC levels (Supplementary Fig. 8b, c), and promoted a nearly 50% higher C2C12 myoblast fusion (Supplementary Fig. 8d), in line with previous observations^34–36^. Similarly, SB431542 added to CM of control FAPs promoted myogenesis of C2C12 cultures (Supplementary Fig. 8d) and to lesser extent of primary myoblasts (Fig. 6d). Importantly, in primary myoblast cultures the negative effects of CM from Osr1cKO FAPs was completely abolished by the addition of SB431542; fusion rate increased to levels of cells treated with control FAPs CM with SB431542 (Fig. 6d). These effects were in essence recapitulated in C2C12 cells, however showing an incomplete rescue of myogenesis in cultures treated with Osr1cKO CM and SB431542 (Supplementary Fig. 8d). These results show that Osr1 regulates the paracrine signaling activity of FAPs both in vivo and in vitro, and that loss of Osr1 leads to inhibition of myogenic differentiation via the TGFβ signaling pathway.

### Osr1-deficient FAPs show a pro-fibrogenic shift with altered ECM expression that affects myogenesis

**Fig. 7:**
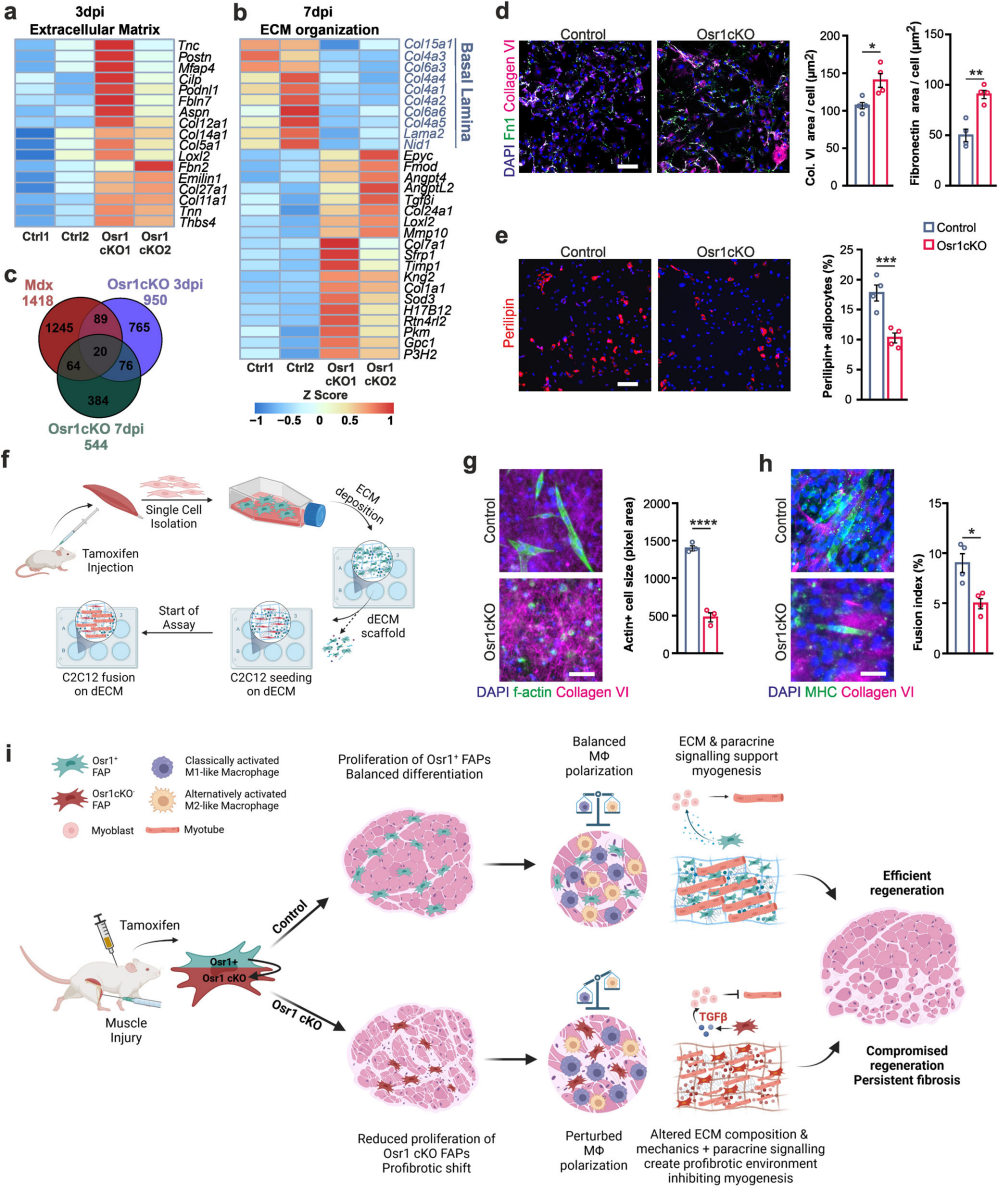
Osr1-deficient FAPs acquire a pro-fibroblastic identity and regulate myogenesis via the ECM. **a**, **b** Heat maps depicting extracellular matrix (ECM) related genes at 3 dpi and 7 dpi in control or Osr1cKO FAPs (*n* = 2). **c** Venn diagram showing overlapping gene deregulation between mdx FAPs and Osr1cKO FAPs at 3 dpi or 7 dpi. **d** Immunolabeling for collagen VI and fibronectin (Fn1) on in vitro recombined FAPs after 6 days of culture. Quantification of the stained areas shown right (*n* = 4). **e** Immunolabeling for Perilipin A/B on in vitro recombined FAPs after 6 days of culture. Quantification of Perilipin+ cells shown right (*n* = 4). **f** Schematic depiction of the in vitro matrix deposition assay. **g** Immunolabeling for Collagen VI and staining for f-actin using phalloidin on C2C12 cells cultured for two days in differentiation medium on dECM from control or Osr1 cells from contralateral hindlimbs of injured 7 dpi animals. Quantification of f-actin+ cell size shown right (*n* = 3). **h** Immunolabeling for Collagen VI and MHC on C2C12 cells cultured for 5 days in differentiation medium on dECM from control or Osr1 in vitro recombined cells. Quantification of fusion index shown right (*n* = 4). **i** Schematic overview of Osr1cKO effects. Data are presented as mean ± SEM; *P*‐value calculated by two‐sided unpaired *t* test; *p < 0.05, **p < 0.01, ****p < 0.0001. *N*‐numbers indicate biological replicates (mice per genotype). Scale bars: 100 μm in (**d**, **e**); 50 μm in (**g**, **h**).

Tissue fibrosis, observed in Osr1cKO muscle, is intimately linked to increased TGFβ signaling^37^. Upregulation of TGFβ target genes *Tgfbi, Scx*, *Col7a1*, *Loxl2* and *Timp1*^38–41^ in 7 dpi Osr1cKO FAPs (Supplementary Fig. 9a) suggested activation of TGFβ signaling. In line, the NIH pathway data source Bioplanet 2019 identified the pathway “TGFβ regulation of extracellular matrix” enriched in genes upregulated in 3 dpi Osr1cKO FAPs (Supplementary Fig. 9b). Consistent with the fibrotic phenotype of Osr1cKO FAPs, ECM-coding genes were upregulated at 3 dpi and 7 dpi (Fig. 7a, b). However, ECM-associated GO terms were also found enriched in downregulated genes at 7 dpi (Fig. 3k). Downregulated ECM genes at 7 dpi encoded proteins of the myofiber basal lamina (Fig. 7b), which is in line with the observed insufficient myofiber formation. Conversely, fibrosis-associated structural ECM- components and modifying enzymes were upregulated Osr1cKO FAPs at 7 dpi. These included known fibrotic markers as *Loxl2* or *Timp1* (Fig. 7b).

Persistent inflammation and fibrosis are hallmarks of muscular dystrophies including Duchenne muscular dystrophy. In line, comparing our Osr1cKO FAP to mdx FAP transcriptome data^42^ revealed 173 common DE genes shared between mdx and Osr1cKO FAPs (Fig. 7c). GO analysis of shared DE genes highlighted ECM-related terms as “collagen containing extracellular matrix” between mdx FAPs and 3 dpi as well as 7 dpi Osr1cKO FAPs (Supplementary Fig. 9c).

To estimate the relative FAP composition of our bulk RNA-seq, we used the deconvolution algorithm MuSiC^43^ on the annotated single cell dataset of regenerating muscle^26^. Deconvolution indicated a phenotypic transcriptional shift of Osr1cKO FAPs towards the tenocyte cluster (Supplementary Fig. 9d) in line with upregulation of tendon- and osteo-chondrogenic transcripts at 3 and 7 dpi (Supplementary Fig. 9e, f). Remarkably, the “*Osr1* cluster” defined by Oprescu et al. mainly expressing genes related to the myofiber basal lamina and secreted signaling molecules^26^ almost disappeared in the Osr1cKO FAP population (Supplementary Fig. 9d). In line, half of the top 51 transcripts from this cluster were downregulated in Osr1cKO FAPs (Supplementary Fig. 9g).

In agreement with increased collagen VI deposition in vivo (Supplementary Fig. 3a), in vitro recombined Osr1cKO FAPs showed higher collagen type VI and fibronectin secretion (Fig. 7d) confirming increased fibrogenic differentiation. Conversely, spontaneous adipogenic differentiation was decreased in Osr1cKO FAPs compared to controls (Fig. 7e). To investigate the direct effects of altered ECM deposition on myogenesis, FAPs were freshly isolated from contralateral muscles of injured control and Osr1cKO animals. Cells were cultured for 21 days to enable the deposition of a coherent ECM (Fig. 7f, Supplementary Fig. 10). This in vitro formed ECM was subsequently decellularized (dECM) and repopulated with C2C12 myoblast. C2C12 cells cultivated on control dECM for 2 days under differentiation conditions showed a spindle-shaped morphology, while cells on the Osr1cKO dECM remained circular and failed to align and spread (Fig. 7g). Assessing the fusion rate of C2C12 cells on control dECM revealed large multinucleated myotubes after 5 days of culture (Fig. 7h). In contrast, myogenic differentiation of C2C12 cells cultured on Osr1cKO dECM was strongly impaired (Fig. 7h). This indicates that loss of Osr1 in FAPs induces a fibrogenic transcriptional shift in part resembling dystrophic FAPs, resulting in aberrant ECM deposition compromising myogenic differentiation.

## Discussion

Our results demonstrate an essential role of the transcription factor Osr1 in the regenerative function of FAPs during muscle healing. Depletion of Osr1 resulted in a perturbed inflammatory response, persistent fibrosis, altered mechanical properties of the transient ECM, impaired timely resolution of degenerated tissue, impaired MuSC activation, and ultimately delayed formation and maturation of new muscle fibers (Fig. 7i).

We note that, due to the use of the ubiquitous CAGG-CreERt line, a possible function of Osr1 in other cells could only be formally excluded by cell type specific inactivation. However, RT-qPCR analysis suggests if at all minimal expression of Osr1 in cells other than FAPs, and several of the in vivo features were recapitulated in an in vitro system where Osr1 was inactivated only in FAPs.

Regulation of FAP proliferation and elimination is essential for muscle regeneration and prevents fibrosis^14,21,42,44–48^. Loss of Osr1 reduced initial FAP pool expansion in a cell-autonomous manner. Pharmacological inhibition of FAP expansion or genetically induced depletion of FAPs resulted in impaired muscle regeneration and fibrosis^15,16^, in part overlapping our model. However, the amount of FAP cells was only slightly reduced at 3 dpi in the Osr1cKO mice and their levels were comparable to controls at 7 dpi. This suggests that reduction of FAP numbers may have a neglectable contribution to delayed healing in our model, but that loss of Osr1 compromises FAP function comparable to a loss of FAP cells.

Conditioned medium experiments confirmed previous observations of FAPs supportive role in myogenesis^3,11^; we, however, note that this effect was only clearly observed when primary myoblasts were used. Our in vitro data show that Osr1-deficient FAPs switch from a beneficial pro-myogenic phenotype to a detrimental state actively suppressing myogenesis. Recent single-cell studies have identified distinct FAP subpopulations that either coexist or represent temporally controlled cellular phenotypes during regeneration^26,49–52^. In addition, tendon progenitors have been defined in muscle as a cell type closely related to FAPs^49,50^. Loss of Osr1 shifts the phenotype of the FAP population toward a more fibrotic, tendon- or cartilage-like profile, overlapping our previous observations in developmental FAP-like cells^24^. Moreover, deconvolution analysis indicated a reduction in the representation of a single cell cluster defined by the expression of *Osr1* itself as a hallmark gene^26^. This suggests that Osr1 is a key regulator of a group of genes defining a specific FAP subtype or state, and prevents FAPs from acquiring alternative fibroblastic states such as tenocyte. Moreover, loss of Osr1 promotes osteo-chondrogenic gene expression in FAPs, reminiscent of osteo-chondrogenic FAP fate in heterotopic ossification^53,54^.

The *Osr1* + cluster contains genes encoding proteins of the myofiber basal lamina, but also secreted signaling molecules^26^, many of which are involved in regulation of immune responses. This suggests a role for the *Osr1* + FAP population in myofiber maturation and immunomodulation. Skeletal muscle regeneration starts with an inflammatory phase, characterized by immune cell infiltration, locally elevated levels of pro-inflammatory cytokines, and active degeneration of existing muscle fibers^1,55^. Neutrophils are the first immune cells to arrive in the regenerative region^7^. Following this, the predominant immune cells are macrophages, which are first activated towards a pro-inflammatory phenotype. These classically activated M1-like macrophages continuously shift their profile to an alternatively activated M2-like restorative phenotype classically considered anti-inflammatory, resolving inflammation, promoting MuSC differentiation, FAP survival and matrix remodeling^9,22,48,56^. The timely resolution of the acute inflammation is essential for subsequent healing stages, which restores tissue structure and function^57^. Our data provide first evidence that impaired FAP function is associated with altered macrophage polarization in vivo. While Osr1 deletion did not affect macrophage infiltration and activation in the injured region at 3 dpi, increased classically activated M1-like relative to alternatively activated M2-like macrophage polarization was seen. To what extent this shift is due to differential recruitment, proliferation, or local activation over time remains to be tested. Several of the cytokines downregulated in Osr1cKO FAPs have been involved in macrophage polarization, suggesting direct crosstalk. CCL2 via its receptor CCR2 induces anti-inflammatory polarization^58^ and inhibits pro-inflammatory cytokine production^59^, and both CCL8^60^ and CCL11 (eotaxin)^61^ recruit alternatively activated M2-like macrophages in cancer metastasis. Collectively, these data suggest that Osr1+ FAPs play an important immunomodulatory role in the early phase of healing and that loss of Osr1 expression may lead to a pro-inflammatory environment.

Exacerbated inflammation, in turn, has undesirable consequences for the function of FAPs and MuSCs. For example, increased TNF-α and NF-κB signaling inhibits MuSC differentiation by suppressing *Myod1* expression^62^. Furthermore, classically activated M1-like macrophages limit FAP expansion via TNFα-mediated apoptosis^22^. However, increased apoptosis of FAPs was also evident in vitro, independent of macrophages. Thus, it is unlikely that altered macrophage polarization drives increased FAP apoptosis we observed in Osr1cKO muscle. Genes involved in the TNF-α signaling cascade were in fact largely downregulated in Osr1cKO FAPs, suggesting that Osr1-deficient FAPs may be less responsive to TNF-induced apoptosis. This might in turn explain their persistence during later phases of regeneration, causing increased ECM deposition and fibrosis.

FAPs are the major producers of a pro-regenerative transient ECM during regeneration^50,63^, while an Osr1cKO FAP-derived ECM inhibited myogenesis in vitro. Osr1cKO FAPs showed a pronounced shift in ECM gene expression characterized by downregulation of genes associated with myofiber basal lamina and the upregulation of several ECM molecules that are usually not expressed, or only to a minor extent, in skeletal muscles. The latter includes the expression of *Col5a1*, *Col11a1*, *Col12a1*, *Tnc* and *Postn*, but also ECM- or signaling-related genes associated with a pro-inflammatory environment, such as Fibromodulin (*Fmod*)^64,65^, Angiopoietin-like 4^66^, angiopoietin-like 2^67,68^ or Secreted Frizzled-related protein 1 (*Sfrp1*)^69,70^. Our in vivo data showed that this altered expression of ECM components coincides with altered mechanical properties of regenerating Osr1cKO muscles. While wild-type muscles show a transient increase in tissue stiffness during healing, Osr1cKO muscles showed significant tissue softening. Aberrant ECM deposition and tissue stiffness could be a direct cause for the observed delay in myofiber formation and maturation, as cells sense ECM composition but also rigidity^71^. Increased matrix stiffness is beneficial for the proliferation and differentiation of MuSCs^63,72–74^. Conversely, decreased stiffness is associated with the formation of fibrotic scar tissue during impaired muscle regeneration^63^, various pathologies^28,75–77^, or aging^78,79^. This altogether suggests that Osr1cKO FAPs produce an inadequate fibrotic replacement matrix during the early phase of regeneration directly interfering with myogenesis.

Impaired muscle regeneration and fibrosis in disease or aging are closely linked to increased TGFβ signaling^30,80–82^. Moreover, in muscle dystrophy, asynchronous waves of inflammation lead to a chronic TGFβ rich environment^48,83,84^. Fibrosis in mdx mice can be exacerbated by injection of TGFβ, and application of TGFβ concomitant to injury in wild type mice resulted in accumulation of fibrotic ECM^85^ comparable to our model. Osr1-deficient FAPs showed increased expression of *Tgfb1*, and we show that the myogenesis-inhibiting effect of Osr1cKO FAPs can be counteracted by blocking TGFβ signaling with a receptor kinase inhibitor. This is in line with previous reports showing a similar anti-myogenic effect of enhanced TGFβ signaling in muscular dystrophies or during regeneration^36,86,87^. Of note, increased TGFβ signaling in the Osr1cKO muscle microenvironment may at the same time impair myogenesis, and also in an autocrine fashion promote the pro-fibrogenic phenotype of FAPs contributing to persistent fibrosis. This is supported by the enrichment of TGFβ pathway-related genes and upregulation of TGFβ pathway downstream targets in the transcriptome of Osr1cKO FAPs. Intriguingly, TGFβ signaling is intimately linked to altered ECM production. Latent TGFβ1 is stored in the ECM, and ECM mechanical properties determine its release and activation^88^. To which extent ECM-derived TGFβ contributes to fibrosis in our model will be an interesting avenue to follow.

In conclusion, our studies show that FAPs are key mediators of an intricate balance coordinating inflammation and regenerative myogenesis, and that Osr1 is an essential transcriptional regulator of the pro-regenerative FAP phenotype.

## Methods

### Mice

Mouse lines were maintained in an enclosed, pathogen-free facility. All experiments were performed in accordance with the European Union legislation for the protection of animals used for scientific purposes, and approved by the Landesamt für Gesundheit und Soziales Berlin under license numbers ZH120, G0114/14 and G0198/19. CAGG-CreER mice were described before^89^. Osr1^flox^ mice were derived from an *Osr1* multifunctional allele (Osr1^MFA^^25^) by crossing with ubiquitous flippase mice^90^. Animals were euthanized using a GasDocUnit®(Medres Medical Research) followed by cervical dislocation.

### Muscle Injury and tamoxifen administration

The tibialis anterior muscle of 4–6 months old mice was injured using the freeze-pierce method. Mice were anaesthetized using isofluoran (Univentor 410 anesthesia unit), or by intraperitoneal injection of 10% (v/v) ketamine/2% (v/v) xylazine (Rompun® 2%) in sterile PBS (5 μl/g body weight), while animals were on a 37 °C heating plate. After that, the skin above the tibialis anterior muscle was opened and the muscle was pierced five times using a syringe needle precooled in liquid nitrogen. On the day before injury until 2 days post-injury, analgesia was performed by subcutaneous injection of Carprofen (5 mg / kg body weight). Tamoxifen (Sigma-Aldrich) was dissolved in a mixture of 90% sunflower oil and 10% ethanol. Animals were injected i. p. with 150 µl of a 20 mg/ml Tamoxifen stock on the day of injury (prior to injury) and on the two following days. As control CAGG-CreER^neg^;Osr1^flox/flox^ or CAGG-CreER^neg^;Osr1^flox/+^ animals were used for histological analysis and stiffness measurements, while CAGG-CreER^+^;Osr1^flox/+^ animals were used as control for experiments involving FACS of Osr1-mGFP+ FAPs (transcriptome analysis and in vitro assays).

### RNA extraction and RT-qPCR

Total RNA isolation was performed using the Direct-zol RNA Microprep kit (for in vitro experiments and FACS-sorted primary cells) and the Direct-zol RNA Miniprep kit (for whole muscle lysate). Reverse transcription for cDNA synthesis was performed using M-MuLV Reverse Transcriptase (Enzymatics, Qiagen) and RNAse Inhibitor (Biotechrabbit). RT-qPCR was performed in a 384-well plate, in a 12 µl mixture consisting of 6 µl Blue SYBR Green mix (Biozym) or GOTaq qPCR Master Mix (Promega), 2 µl primer pairs (2.5 µM each) and 5 µl template of cDNA. RT qPCR was performed in three technical replicates from each of at least three biological replicates (representing individual animals or cells derived from one animal). Analysis was performed using the ABI Prism HT 7900 real-time PCR detection system (Applied Biosystems) equipped with SDS software version 2.4 (ThermoFisher Scientific) or the QuantStudio 7 (Applied Biosystems) with the software version 1.3 (ThermoFischer Scientific). The mean relative values of the three technical replicates were normalized to the values of GAPDH, which was used as a housekeeping gene. To calculate the relative expression level of the respective gene, the double delta Ct (ΔΔCt) method was used. All primers used were purchase from Eurofins Scientific and are listed in Table S1.

### Tissue preparation and histology

Directly upon dissection, muscle was embedded in 6% (w/v) gum tragacanth (Sigma-Aldrich) dissolved in H_2_O, and snap frozen in ice-cold isopentane (precooled in liquid nitrogen, −160 °C). Muscle tissue was sectioned at 10 µm thickness onto Superfrost Plus slides (Thermo Scientific) and stored in −80 °C until use. Hematoxylin and Eosin (Thermo Fisher) (H&E) staining and picrosirius red staining were performed according to standard procedures. Stained samples from *n* = 3 mice per genotype per time point (3, 5, 10,17, and 28 dpi) were imaged in total using a Leica brightfield microscope equipped with an automated XY scanning stage. The area size of the collagenous matrix deposition was quantified in Image J from the picrosirius red staining and was further normalized to the size of the total area size of the whole regenerative region.

### Immunolabelling

Prior to antibody labeling, sections were allowed to reach room temperature (RT) slowly, slides were immersed in PBS for 5 min. and fixed in 4% PFA for 10 min. at RT. Slides were shortly washed in PBS and then permeabilized with 0,4% (v/v) Triton X-100 (Sigma Aldrich) in phosphate buffer (PBS) for 10 min. Sections were blocked with 5% bovine serum albumin (Sigma Aldrich) in 0,1% Triton X-100 in PBS for 1 h at RT. Primary antibodies were diluted in 5% BSA in 0,1% Triton X-100 in PBS and incubated overnight at 4 °C, the following day slides were washed three times in PBS for 5 min. Secondary antibodies were diluted in 5% BSA in PBS and incubated for 1 h at room temperature, followed by three short washes in PBS for 5 min. Nuclei were stained with 5 µg/µl 4′,6-diamidino-2-phenylindole (DAPI; Invitrogen) and slides were mounted with FluoromountG (SouthernBiotech).

For MHC3 immunostaining, antigen retrieval was performed upon fixation. Sections were treated in chilled methanol, kept in −20 °C for 6 min, and subsequently washed in PBS. Then, slides were immersed in 1 mM Ethylendiaminetetraacetic acid (Roth) at 95 °C for 10 min. The slides were left at RT for 30 min and washed in PBS. Blocking and antibody staining was performed as described above.

For Pax7 immunostaining, upon fixation with 4% PFA, slides were permeabilized in chilled methanol for 6 min at RT. Epitope retrieval was performed in boiling 1 mM citric acid pH 6 for 2 min and 30 sec in a microwave. Slides cooled to RT, washed in PBS and blocked in 5% BSA IgG-free (Jackson Immuno Research) in PBS. To decrease background, sections were incubated with anti-mouse IgG Fab fragments (Jackson Immuno Research) diluted in PBS 1:100 for 30 min at RT. Primary and secondary antibody were diluted in 5% BSA IgG free and staining was performed as described above. A list of primary and secondary antibodies is provided in Tables S2 and S3.

For cell immunostaining, isolated cells were added on the coated coverslips (1 h at RT in Poly-L-lysine (Milipore) diluted 1:100 in water) and allowed to adhere for 1 h at 4 °C. Then, cells were fixed in 4% PFA for 15 min at RT and washed shortly in PBS. Samples were permeabilized in 0,4% (v/v) Triton X-100 (Sigma Aldrich) in phosphate buffer (PBS) for 10 min. Primary and secondary antibody labeling followed as described above. Cytospun collected cells were stained following the same procedure. TUNEL staining on cytospun and cultured cells was performed using the DeadEnd^TM^ kit (Promega) according to the manufacturer’s instructions.

### Cell sorting of FAPs and MuSCs by FACS

FAP and MuSC isolation from muscle was performed as described in^25^. Briefly, TA muscle was isolated and roughly minced with a small scissor in high-glucose DMEM medium (PAN Biotech) containing 10% fetal bovine serum (PAN biotech), 1% Penicillin Steptomycin (P/S) solution (PAN Biotech, 10.000 U/ml) and 2,5 mg/ml Collagenase A (Roche) for 45 min at 37 °C with gentle shaking. Muscle lysates were further digested with 2 IU/ml of Dispase II (Sigma Aldrich) diluted in minimum amount of PBS for 30 min. To stop the enzymatic digestion, 10% FCS DMEM was added to the sample and subsequently the sample was passed ten times through a 20 G syringe needle. Cells were filtered first through a 70 µm and then through a 40 µm cell strainer (Fischer Scientific) and collected by centrifugation at 400xg for 10 min. Cells were resuspended in filtered “FACS buffer” containing 1% BSA and 2 mM EDTA, labeled with the following antibodies: anti-CD45-APC, anti-Cd31-APC, anti-TER119-APC, anti-α7-integrin-PE, and anti-Ly6A/e-APC-Cy7 (Table S4) for 30 min on ice and washed three times with FACS buffer prior to sorting. Propidium iodide was used as a viability dye. Cell sorting and analysis was performed on a BD FACSAriaII SORP (BD Biosciences). Single-stained and fluorescence minus-one controls were used for setting the sorting gates. Data were collected using the BD FACSDiva software version 8.0.1.

### Immune cell flow cytometry analysis

Muscles were cut into small pieces and transferred to a gentleMACS C tube (Miltenyi Biotec, Bergisch Gladbach, Germany) containing TrypLE Express Enzyme (ThermoFisher, Waltham, MA, USA) in a 37 °C water bath for 5 min. The sample was run on a gentleMACS Dissociator (Miltenyi Biotec, Bergisch Gladbach, Germany) using the predefined program for murine muscle dissociation. The tube was transferred back to the 37 °C water bath for another 5 min and the dissociation program was repeated. The dissociated tissue was then filtered through a 40 µm nylon mesh (ThermoFisher, Waltham, MA, USA) and thoroughly washed with PBS. Cells were counted and incubated with live-dead stain (LIVE/DEAD Fixable Blue for UV excitation, ThermoFisher, Waltham, MA, USA) at 4 °C. Washing steps were performed using PBS supplemented with 0,5 % w/v bovine serum albumin and 0,1 % sodium acid (both Sigma-Aldrich, St. Louis, MO, USA). Surface marker incubation was performed before intracellular staining at 4 °C. Intracellular staining for epitopes was achieved using the fixation buffer and intracellular permeabilization buffer kit (BioLegend, San Diego, CA, USA). Antibodies are listed in Table S4. Flow analysis was run on a CytoFlex LX system (BeckmanCoulter, Brea, CA, USA) and population gating and tSNE analysis were performed with FlowJo (BD Biosciences, Franklin Lakes, NJ, USA). The gating strategy performed as previously published^91^. Outliers were identified with the ROUT method and excluded from the analysis.

### Isolation of adherent fibroblasts for in vitro FAP culture

Adherent connective tissue fibroblasts that are phenotypically identical to FAPs were isolated from skeletal muscle in essence as described before^31^, thus we refer to these cells as FAPs. For generation of conditioned medium, co-culture assays and for the decellularization assays, cells were isolated from contralateral hindlimbs of injured 7 dpi animals. This was done to reduce animal usage and to achieve a pre-activated “alert” state of FAPs^25,33^. For cell isolation, briefly, the TA, gastrocnemius and the quadriceps muscles were carefully isolated and minced with a scissor. Tissue digestion was performed as for flow cytometry. After centrifugation, cells were resuspended in DMEM with 10% FCS, placed in a 10 cm dish and allowed to attach for 90 min at 37 °C. Supernatant containing non- attaching cells was removed, adherent cells were washed once with PBS and fresh DMEM with 10% FCS was added (Passage 0). After 3-4 days of growth, cells were trypsinized, counted using the automated LUNA^TM^ cell counter (Logos Biosystem), and immediately used for the respective assay (Passage 1). For every experiment, freshly isolated FAPs (Passage 1) were used.

### Isolation of primary myoblasts

Skeletal muscle progenitors were isolated from hindlimbs muscles of 8–10 weeks old mice as described in^92^. Briefly, muscles were finely minced and digested with Collagenase A for 1 h at 37 °C. Cells were centrifuged at 500 g for 10 min and supernatant was removed. Cell pellet was resuspended in full myoblast (MB) proliferation medium containing 20% FCS, 10% HS, 2.5 ng/ml recombinant human FGFb (Gibco), 0.5% chicken embryo extract (ZellBio). The pellet was placed on Matrigel (Corning) coated plates (working concentration of 0.9 mg/ml) where myoblast migration from the minced myofibers was observed on day 3 of culture. At that point, cells were trypsinized and plated for 1 h on type I rat tail collagen (working concentration 0.1 mg/ml) (Corning) coated plates for removal of fibroblastic and non-myogenic cells. Next, medium containing non-attached cells was collected and placed on Matrigel coated plates for expansion of the myoblast culture.

### In vitro recombination of the *Osr1* locus

0.5 µM of 4-hydroxytamoxifen (4-OHT) was added to the medium of passage 0 control or Osr1cKO FAPs. 4-OHT treatment was repeated on day 1 and day 2 of culture, without discarding the old medium. The efficiency of the recombination was measured via genomic qPCR for the exon 2 of *Osr1* and by RT-qPCR. For differentiation assays, cells were trypsinized upon 50–60% confluency to avoid spontaneous differentiation. Approximately 25.000 FAPs were seeded on coverslips in 24 well plates and cultured in DMEM 10% FCS for 6 days. Fresh medium was added every 2 days. Cells were fixed with 4% PFA and immunolabeled as described above.

### Indirect co-culture of FAPs and C2C12/primary myoblasts

For transwell assays, 35.000 control or Osr1cKO FAPs from contralateral hindlimbs of injured 7 dpi animals were seeded in the insert and cultured in transwell plates for 14 days in proliferation medium (high-glucose DMEM, 10% FBS, 1% P/S). Upon confluency, 100.000 C2C12 cells (ATCC) per well were seeded and allowed to attach for 4 h. For primary myoblasts, 180.000 cells were seeded on 0.2% gelatin-coated coverslips and allow to grow confluent in MB proliferation medium. The proliferation medium was then removed and replaced with differentiation medium (high-glucose DMEM, 2% horse serum (PAN biotech), 1% P/S). After 4 days of differentiation, cells were fixed for immunolabeling. To analyze C2C12 fusion, four different areas per sample were analyzed and the number of nuclei in MyHC+ fibers was normalized to the total number of nuclei.

### Conditioned medium assays

30.000 recombined control or Osr1cKO FAPs from contralateral hindlimbs of injured 7 dpi animals were seeded on 24-well plates and expanded in DMEM 10% FCS and 1% P/S until they reached confluency. Cells were briefly washed with PBS, then 300 µl of DMEM 1% P/S without FCS was added. After 24 h conditioned medium (CM) was collected, spun for 15 min in 4 °C at 3.000 rcf and supernatant was isolated. CM was stored in −80 °C and thawed on ice before use.

For CM treatment, 50.000 C2C12 cells were seeded on coverslips in 24-well plates and kept in proliferation medium (high-glucose DMEM, 10% FCS, 1% P/S) for 3 days. Conditioned medium (500 µl) was supplemented with 2% HS and used as differentiation medium for the C2C12 cells. As positive control, fresh differentiation medium (high-glucose DMEM, 2% horse serum, 1% P/S) was used. New conditioned medium was added on cells on day 2, and on day 4 cells were fixed with 4% PFA and immunolabeled as described above. Fusion index was calculated as the ratio of nuclei in MHC positive fibers versus the total number of nuclei. Four different areas per sample were imaged and quantified.

For SB431542 treatment, 20.000 C2C12 cells per well were seeded on an 18-well IBIDI μ slide in proliferation medium. In the case of primary myoblasts, the ibidi angiogenesis plate was coated with Matrigel and 40.000 cells were seeded per well. On the following day, cells were starved for 5 h in high-glucose DMEM with 0% FCS and then cells were pre-treated for 1 h with 0.5 μM SB431542 (Selleck chemicals). SB431542 medium was aspirated, cells were briefly washed with PBS. Then, 80 µl of conditioned medium supplemented with 2% HS and 0.5 μM SB431542 was added to the cells. On day 3 of differentiation, cells were fixed and immunolabeled as described above. Each chamber of the slide was completely imaged, fusion index was quantified as described above.

### Decellularization and dECM assays

To generate the dECM scaffolds, 30.000 recombined control or Osr1cKO FAPs from contralateral hindlimbs of injured 7 dpi animals were seeded on 0.1% gelatin (Roth) coated coverslips in 24-well plates. Cells were cultured in matrix medium consisting of high-glucose DMEM, 10% FCS, 1% P/S and 50 µM of ascorbic acid. Matrix medium was changed every 2–3 days. Cells were cultured for approximately two weeks until a visible thin layer of matrix formed, after which cells were removed using the prewarmed 0,5% Triton-X-100 and 20 mM NH_4_OH in PBS. Afterwards the three-dimensional matrices were gently washed with PBS and 100.000 C2C12 were seeded in differentiation medium (high-glucose DMEM, 2% horse serum (Pan Biotech), 1% P/S) for 2 or for 5 days. Half of the medium was then aspirated and 4% PFA was added for 15 min. at RT. Attached cells and ECM scaffolds were immunolabeled as described above.

### Nanoindentation

The surface elasticity of 15 µm sections from injured TA muscles was measured using the Piuma nanoindenter (Optics11life). The system was calibrated for Young’s modulus measurement, approaching the slide surface and performing an initial wavelength scan. The cantilevers used had a tip radius in the range of 9.5 µm and stiffness of 0.52 N/m. Tissues were immersed in deionized water for 10 min prior to the measurements. Matrix scans of 10 × 10 were performed setting the step size of the cantilever to 15 µm. The data were acquired using the Piuma software.

### RNA sequencing

GFP + FAPs were isolated via FACS from injured muscles from 2 Controls and 2 Osr1 cKO animals at 3 dpi and from 8 Controls and 8 Osr1cKO animals at 7 dpi. 1 sample each at 3 dpi, and 4 pooled samples at 7 dpi served as a biological replicate. RNA was isolated using the Micro RNA Kit (Zymo Research), the RNA concentration was measured using a QubitFluorometer (Invitrogen), and the quality of the RNA yield was measured with the Bioanalyzer 2100 (Agilent). After quality control using Agilent’s Bioanalyzer sequencing libraries were prepared from 100 ng of total RNA per sample following Roche’s stranded “KAPA RNA HyperPrep” library preparation protocol for dual indexed Illumina libraries: First the polyA-RNA fraction was enriched using oligo-dT-probed paramagnetic beads. Enriched RNA was heat-fragmented and subjected to first strand synthesis using random priming. The second strand was synthesized incorporating dUTP instead of dTTP to preserve strand information. After A-tailing Illumina sequencing compatible unique dual index adapters were ligated. Following bead-based clean-up steps the libraries were amplified using 11-12 cycles of PCR. Library quality and size were checked with qBit, Agilent Bioanalyzer and qPCR. Sequencing was carried out on an Illumina HiSeq 4000 system in PE75bp mode and on NovaSeq4000 in PE100bp mode, respectively. Read mapping to the mouse genome (mm10) was performed using STAR in the Galaxy Europe platform, and differential gene expression analysis was performed using DESeq2. Genes were considered as being differentially expressed if the fold-change of KO vs Control was greater than 1.2, if the p-value was below 0.05 for the 3 dpi samples and if the Benjamini-Hochberg adjusted p-value (padj) was below 0.1 for the 7 dpi samples. Transcripts per million (TPM) abundances were calculated from the mean normalized fragment counts given by DESeq2 for all samples. Gene ontology and pathway analysis was performed using the functional annotation tools Enrichr^93^ and g:Profiler^94^.

### Immunoblotting

Protein isolation was performed upon cell sample homogenization using RIPA buffer (50 mM Tris‐Hcl, pH 8.0; 150 mM Nacl; 1% NP‐40; 0.5% Sodium deoxycholate; 0.1% SDS). Protein concentration was determined using the Pierce BCA Protein Assay Kit (Thermo Fischer #23225). Total protein was loaded and separated in SDS-page gels and then transferred to PVDF membrane (GE Healthcare). For blocking, 5% BSA in TBST was applied on the membrane for 1 h at RT. Primary antibodies were diluted in the blocking buffer and were incubated overnight at 4 °C. Membranes were washed three times in PBST, and then incubated with HRP-conjugated secondary antibodies diluted in PBS for 1 h at RT. Antibodies are listed in Table S2. The Fusion X spectra gel documentation system (Vilber) was used for image acquisition (FUSION FX software).

### Reporting summary

Further information on research design is available in the Nature Research Reporting Summary linked to this article.

## Supplementary information

Supplementary Material

Reporting Summary

## Data Availability

All sequencing data are available via the BioProject accession number PRJNA938360 and GEO accession number GSE226683.
