# Supplementary material for: Odd skipped-related 1 controls the pro-regenerative response of fibro-adipogenic progenitors
Source: NPJ Regen Med. 2023 Apr 5;8:19. doi: 10.1038/s41536-023-00291-6 (PMC10076435; doi:10.1038/s41536-023-00291-6)
Supplement: Supplementary file 1 — Supplementary Material [file 41536_2023_291_MOESM1_ESM.pdf]

Odd skipped-related 1 controls the pro-regenerative response of Fibro-Adipogenic Progenitors

Georgios Kotsaris, Taimoor H. Qazi, Christian H. Bucher, Hafsa Zahid, Sophie Pöhle-Kronawitter, Vladimir Ugorets, William Jarassier, Stefan Börno, Bernd Timmermann, Claudia Giesecke-Thiel, Aris N. Economides, Fabien Le Grand, Pedro Vallecillo-García, Petra Knaus, Sven Geissler and Sigmar Stricker

Supplementary Figures and Tables

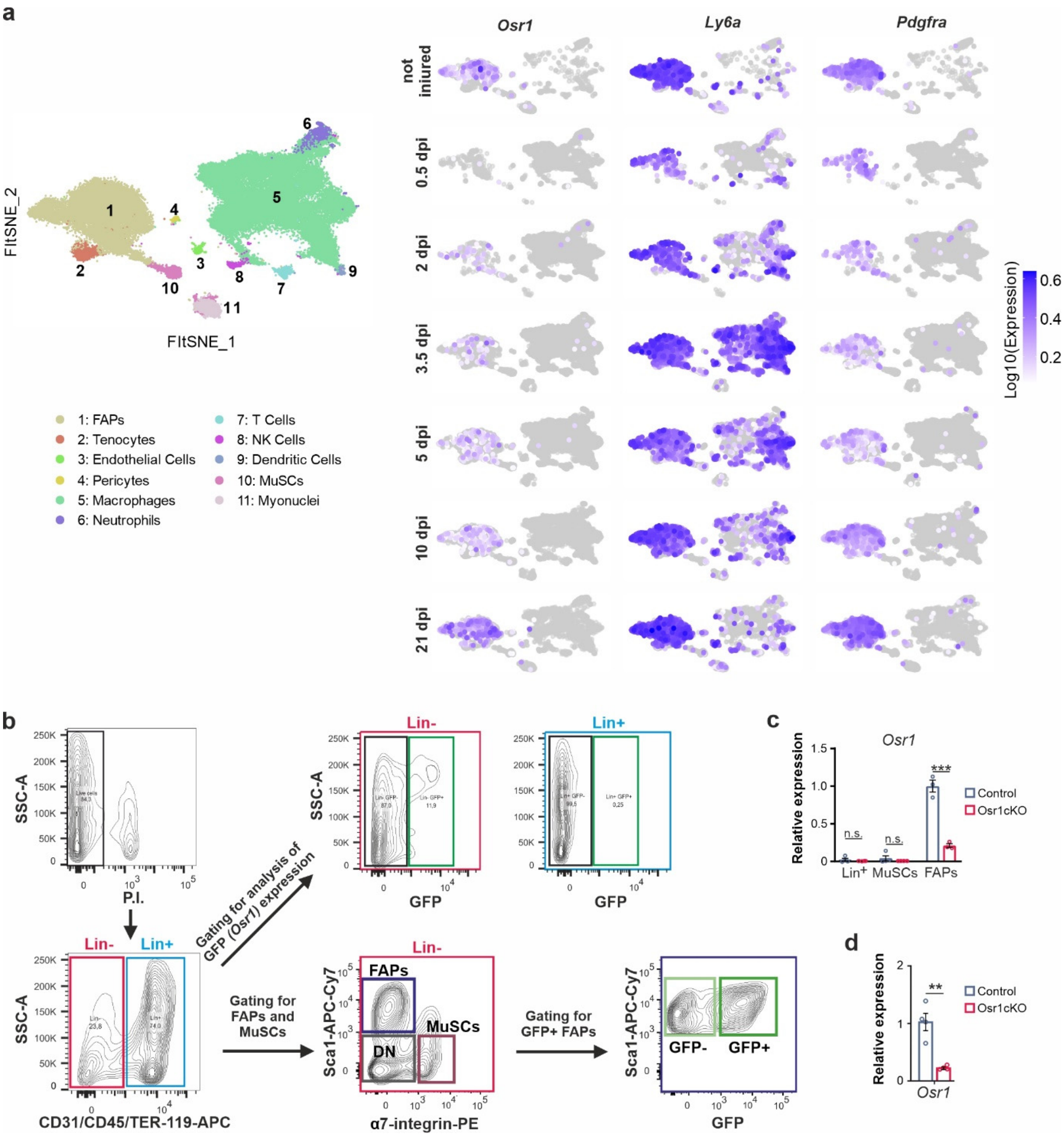

Supplementary Figure 1 Efficacy of Osr1 conditional inactivation and specificity of Osr1 expression.

a Analysis of *Osr1* expression in comparison to *Pdgfra* and *Ly6a* (Sca-1) in single cell data from Oprescu et al. of uninjured muscle and of indicated time points post injury. Cluster annotation shown left. **b** FACS sorting strategy for isolating propidium iodide (P.I.)-low live cells, followed by gating for Lin+ (CD31+ CD45+ TER-119+) and Lin- (CD31- CD45- TER-119-) cells. Top right panel: Analysis of GFP expression from the recombined Osr1 locus in Lin- and Lin+ cells from 3 dpi muscle. Note no GFP+ cells were detected in Lin+ hematopoietic and endothelial cells. Bottom right panel: FACS sorting strategy for FAPs and MuSCs as used for Fig. 3b, c, d; Fig. 5b-h; Fig. 6b, d; Fig. 7g, h. Right: isolation of GFP+ FAPs as used for RNA Sequencing. **c** RT-qPCR analysis of *Osr1* mRNA expression in FACS-isolated FAPs, MuSCs and Lin+ cells from control and Osr1cKO mice at 3 dpi (n=3). **d** RT-qPCR analysis of *Osr1* mRNA expression in whole muscle tissue from control and Osr1cKO mice at 3 dpi (n=4). Data are mean ± SEM; P-value calculated by two-sided unpaired t-test; $**p < 0.01$. N-numbers indicate biological replicates (mice per genotype).

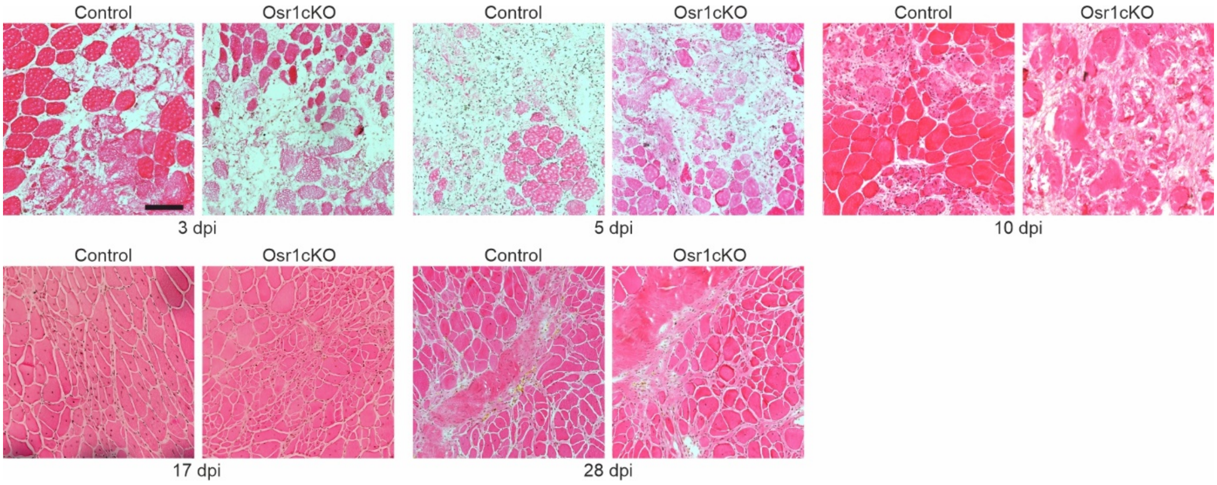

Supplementary Figure 2 Delayed muscle regeneration and fibrotic appearance of Osr1cKO regenerating muscle.

Hematoxylin and eosin staining of control and Osr1cKO muscle sections at indicated days post injury (dpi). Scale bar: 100 µm.

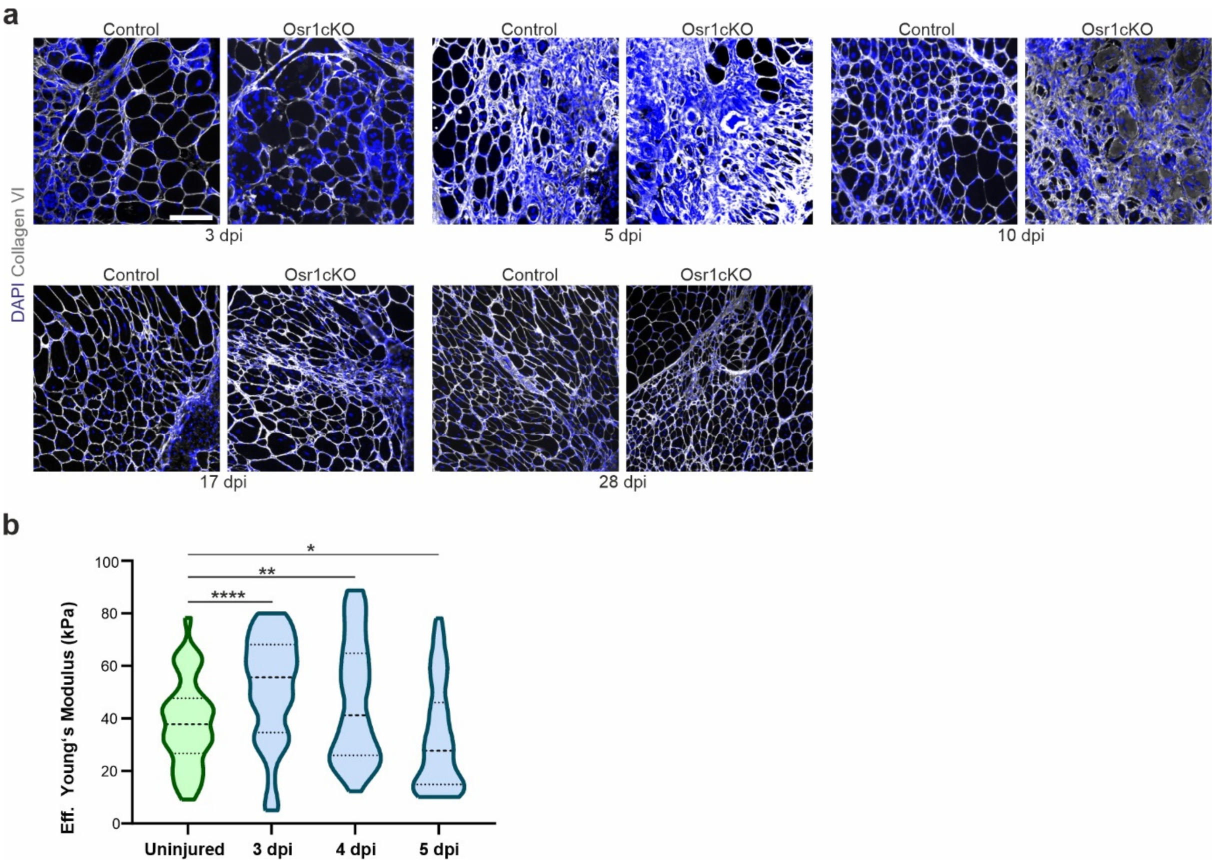

Supplementary Figure 3 Fibrotic appearance of Osr1cKO regenerating muscle.

a Immunolabeling for Collagen VI on control and Osr1cKO muscle sections at indicated dpi. **b** Tissue stiffness measurement assessed by nanoindentation of wild type regenerating muscle tissue sections at indicated dpi (n=3). Data are mean ± SEM; P-value calculated by two-sided unpaired t-test; * $p < 0.05$, ** $p < 0.01$, **** $p < 0.0001$. N-numbers indicate biological replicates (mice per genotype). Scale bar: 100 μm.

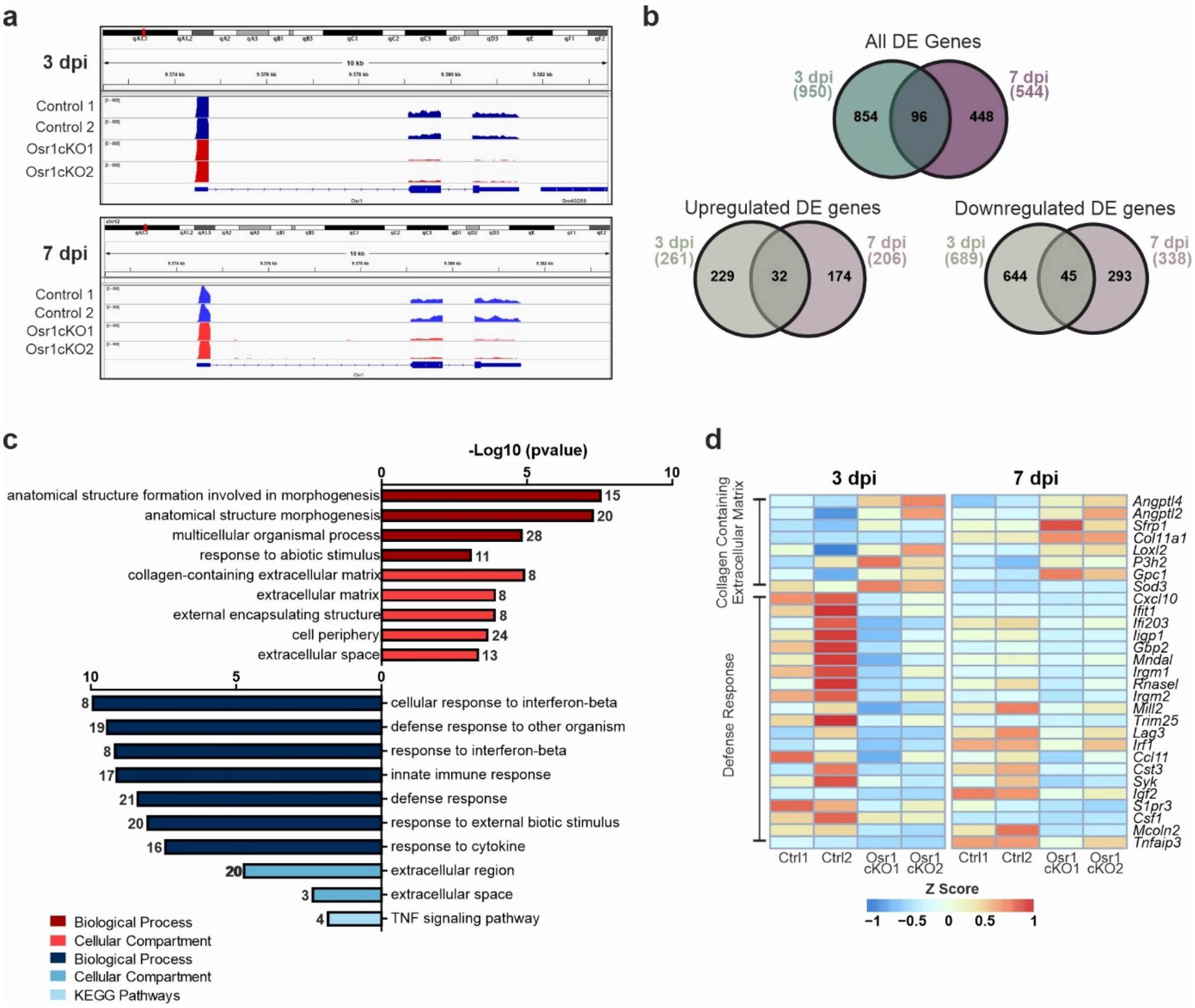

Supplementary Figure 4 Supplementary transcriptome analysis of Osr1cKO FAPs.

a Genome browser view of the *Osr1* locus with RNA seq data from 3 and 7 dpi. Note efficient recombination indicated by strongly reduced exon 2 and 3 reads. **b** Venn diagram depicting common regulated genes between the 3 and the 7 dpi Osr1cKO FAPs. **c** GO term analysis of genes commonly upregulated (top) and downregulated (bottom) in Osr1cKO FAPs at 3 and 7 dpi. **d** Heat maps showing genes belonging to the GO terms “Collagen-containing extracellular matrix” and “Defense response” at 3 and 7 dpi showing continuous deregulation.

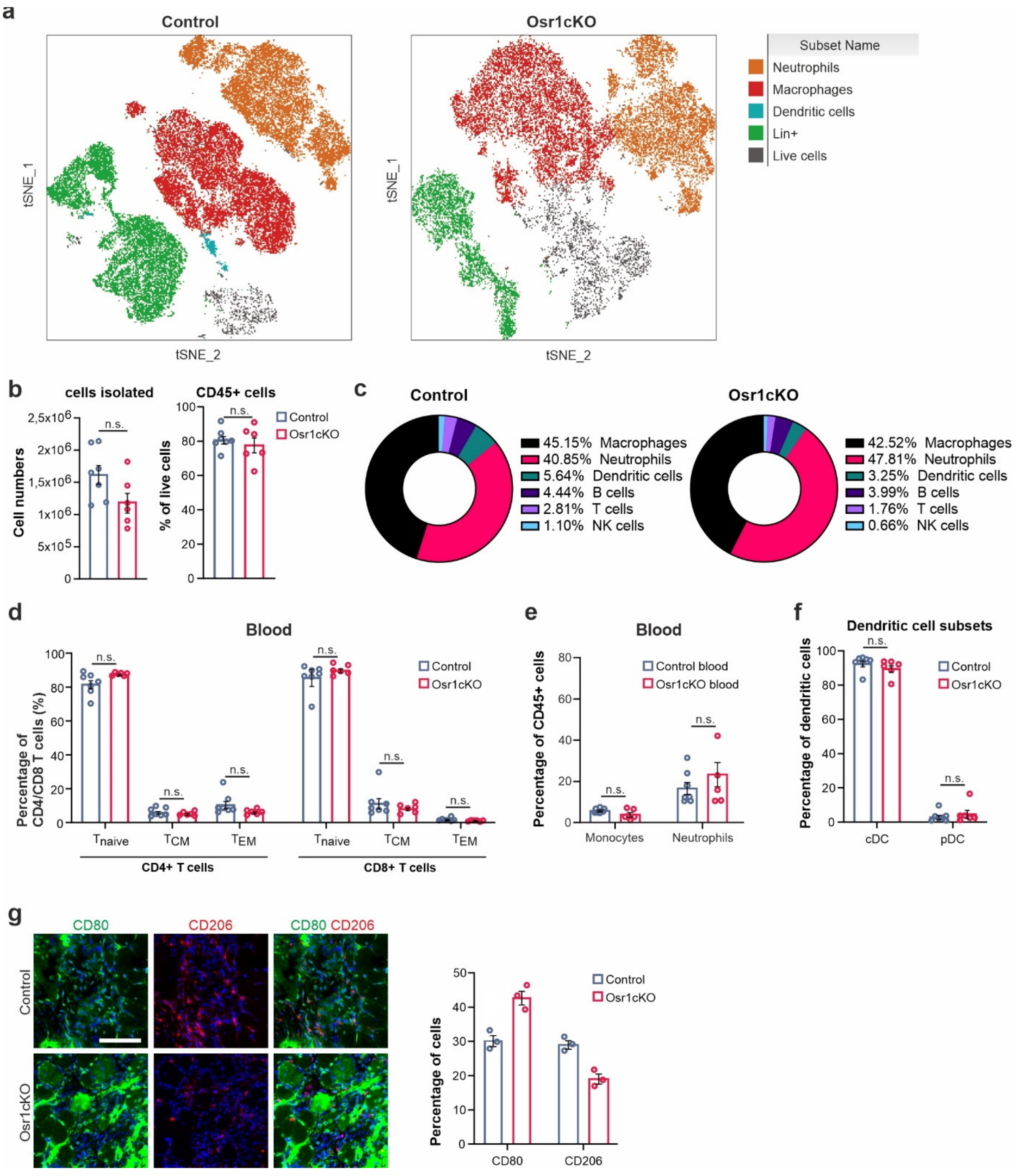

Supplementary Figure 5 Supplementary data immune cell profiling at 3 dpi.

a tSNE plot depiction of immune cell populations identified in 3 dpi control or Osr1cKO muscle. Lin+ is defined CD3+, CD19+, CD335+. **b** Flow cytometry quantification of all live cells analyzed and percentage of CD45+ cells in 3 dpi control or Osr1cKO muscle. **c** Percentages of immune cell populations identified in control and Osr1cKO muscle. **d** Flow cytometry quantification of T-cell cell subsets in blood of 3 dpi control or Osr1cKO mice. **e** Flow cytometry quantification of macrophages and neutrophils in blood of 3 dpi control and Osr1cKO mice. **f** Flow cytometry quantification of Dendritic cell subsets in 3 dpi control or Osr1cKO muscle. **g** Immunolabeling for CD80 and CD206 on tissue sections of 3 dpi control and Osr1cKO animals. Relative quantification of CD80+ vs. CD206+ cells is shown right. In b - f n=7 for control and n=6 for Osr1 cKO. In g n=3 for both groups. Data are mean ± SEM; P-value calculated by Mann-Whitney test; N-numbers indicate biological replicates (mice per genotype). Scale bar: 100 µm.

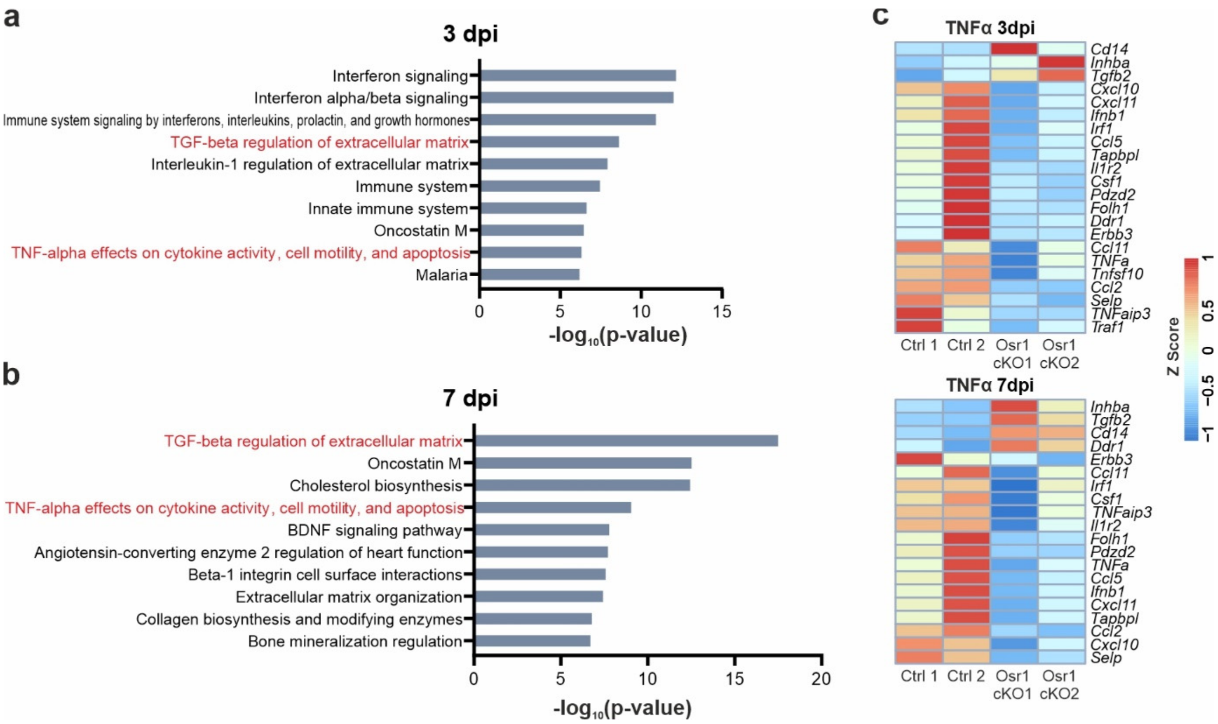

Supplementary Figure 6 TNFα pathway analysis in transcriptome data of Osr1cKO FAPs

a, b GO term analysis of DE genes in Osr1cKO FAPs relative to control FAPs at 3 dpi (top) and 7 dpi (bottom). **c** Heat maps showing downregulation of TNFα pathway genes belonging to the highlighted terms in Osr1cKO FAPs.

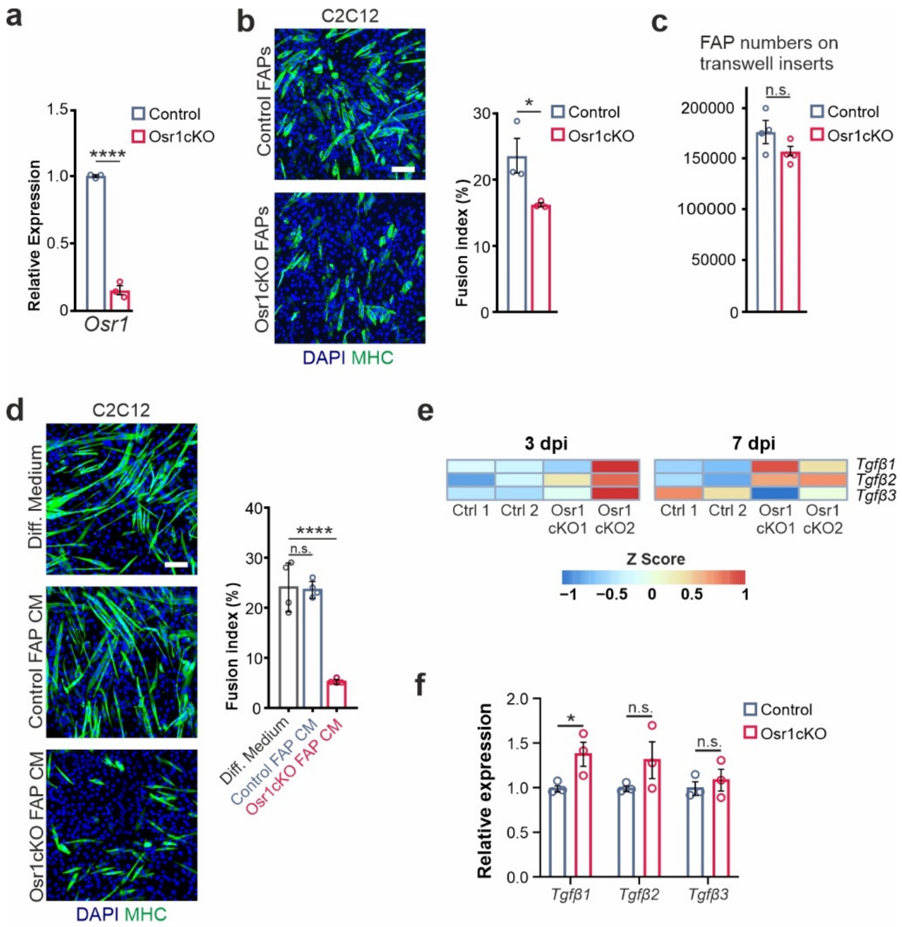

Supplementary Figure 7 Effects of FAP coculture and FAP conditioned medium on C2C12 cells, and upregulation of *Tgfb* genes in Osr1cKO FAPs.

a RT-qPCR analysis of Osr1 mRNA expression in FAPs isolated at 7 dpi from contralateral muscle of injured control and Osr1cKO mice (n=3). **b** Immunolabeling for MHC to detect myotube formation from C2C12 cells co-cultured with control or Osr1cKO FAPs; quantification of fusion index is shown right (n=3). **c** Quantification of FAP numbers in transwell plates at the day of analysis**. d** Immunolabeling for MHC to detect myotube formation from C2C12 cells in differentiation medium or differentiation medium supplemented with control or Osr1cKO CM; quantification of fusion index is shown right (n=4). **e** Heat maps showing *Tgfb1,2* and *3* gene expression in control or Osr1cKO FAPs at 3 and 7 dpi. **f** RT-qPCR analysis of *Tgfb1,2* and *3* gene expression in *in vitro* recombined Osr1cKO FAPs (n=3). Data are mean ± SEM; P-value calculated by two-sided unpaired t-test; * $p < 0.05$, **** $p < 0.0001$. N-numbers indicate biological replicates (mice per genotype). Scale bars: 100 µm.

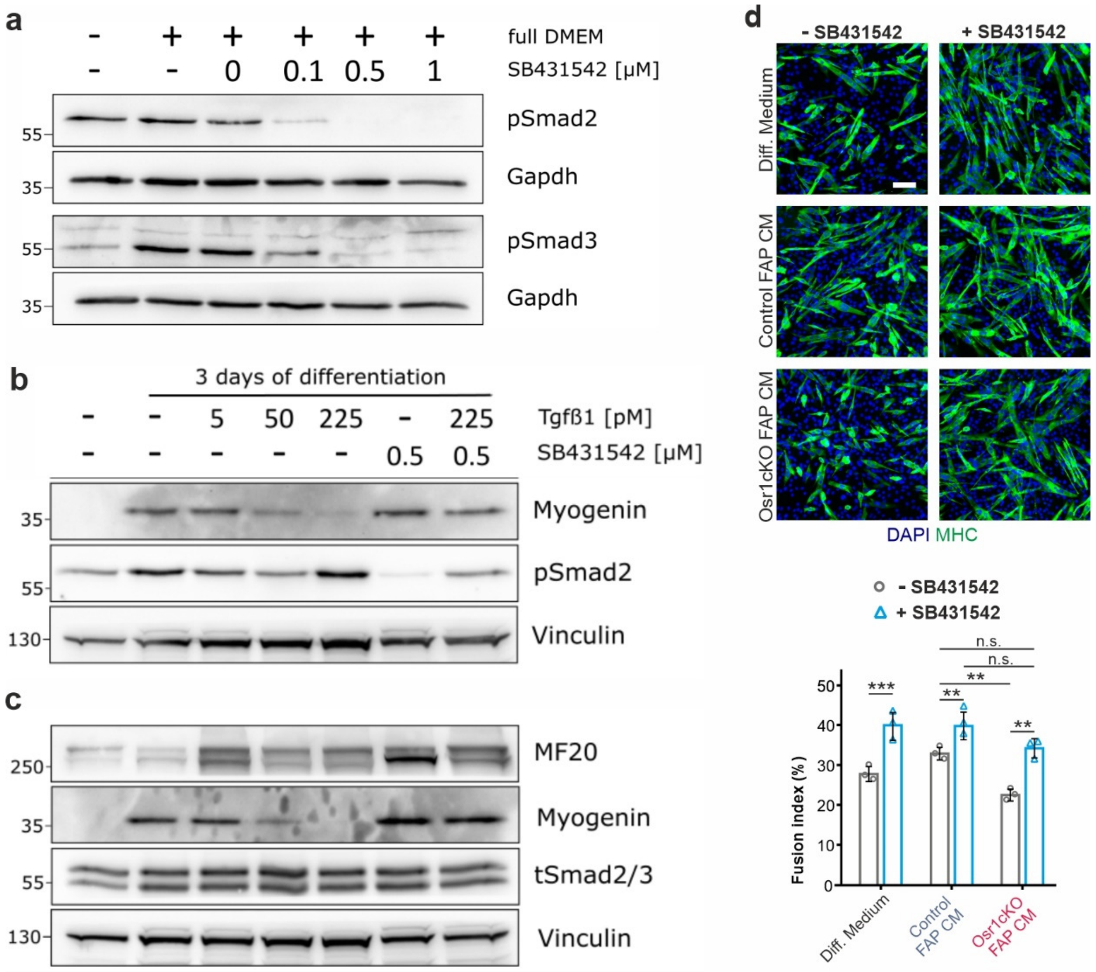

Supplementary Figure 8 Efficacy of TGFβ pathway inhibition and effects of SB431542 in C2C12 cells.

a Western blot analysis of TGFβ pathway activity in C2C12 cells cultured in DMEM and treated with increasing concentrations of SB4315242 assessed by detection of phosphor-Smad2 phosphor-Smad3. **b, c** Western blot analysis of phospho-Smad2, Myogenin and myosin heavy chain (MF20) expression in C2C12 cells treated with recombinant TGFβ1 and SB4315242. All blots of each panel are derived from the same experiment, **d** Immunolabeling for MHC to detect myotube formation from C2C12 cells in differentiation medium or differentiation medium supplemented with control or Osr1cKO CM, with or without TGFβ pathway inhibitor SB431542; quantification of fusion index is shown right (n=3).Data are mean ± SEM; P-value calculated by ANOVA; ** $p < 0.01$, *** $p < 0.001$. N-numbers indicate biological replicates (mice per genotype). Scale bar: 100 µm.

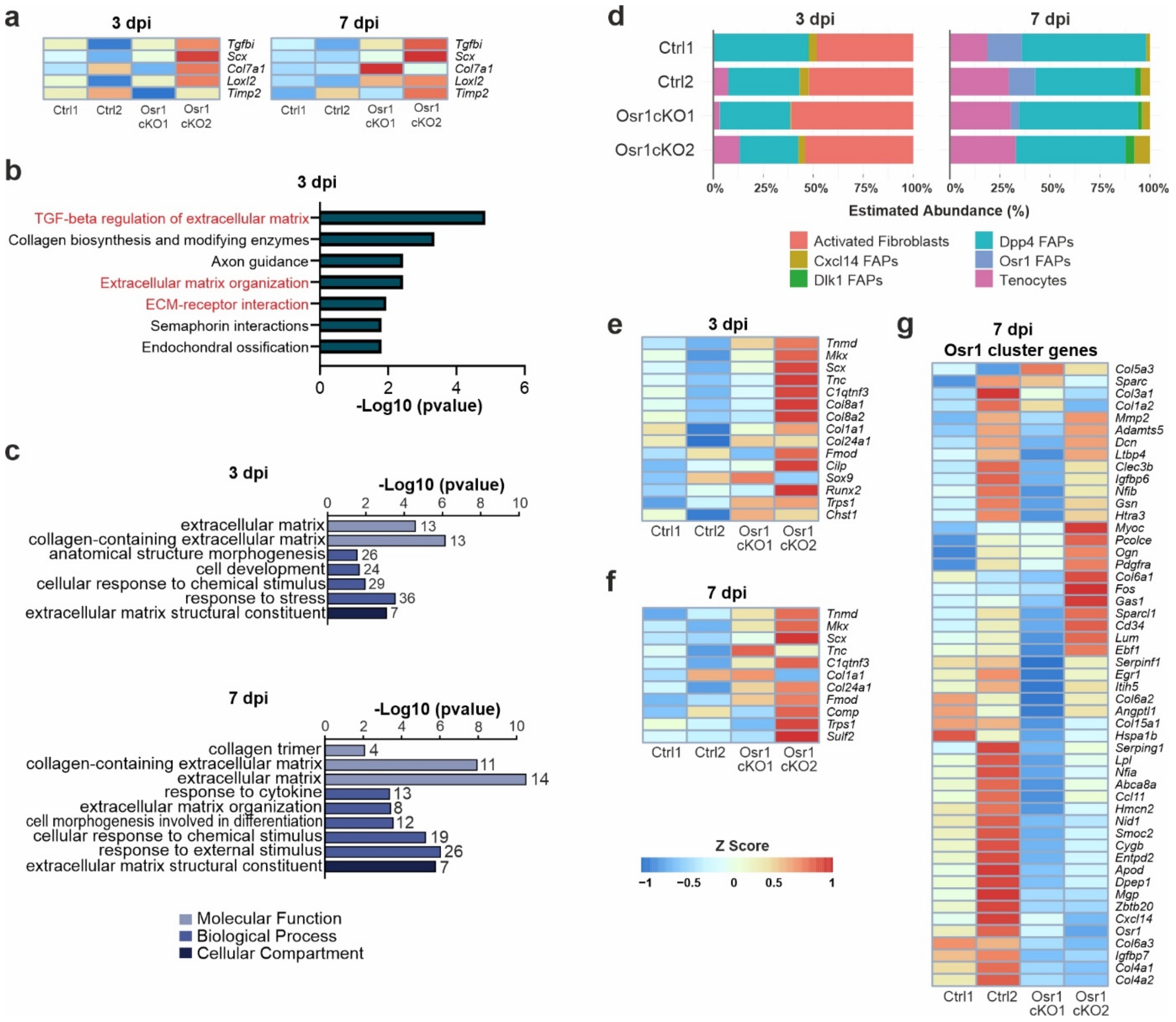

Supplementary Figure 9 Transcriptional fibrogenic shift of Osr1cKO FAPs.

a Heat maps showing TGFβ target gene expression in control or Osr1cKO FAPs at 3 and 7 dpi. **b** Bio planet 2019 pathway analysis of genes upregulated in 7 dpi Osr1cKO FAPs relative to controls. **c** GO terms analysis of the common deregulated genes between mdx FAPs and the 3 or 7 dpi Osr1cKO FAPs. **d** Deconvolution analysis of control and Osr1cKO FAP bulk transcriptome data on single cell sequencing data from Oprescu et al. **e, f** Heat maps showing upregulation of tendon- and cartilage-associated genes in Osr1cKO FAPs relative to controls. **g** Heat map depiction of signature genes characterizing the "*Osr1*" cluster in Oprescu et al.

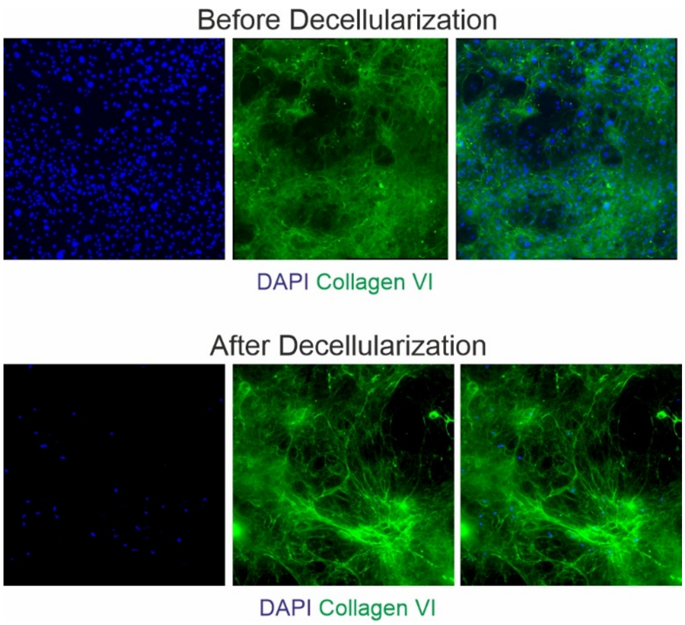

Supplementary Figure 10 Efficiency of in vitro ECM deposition and the decellularization.

Supplementary Tables

Supplementary Table 1 Primers for RT-qPCR

	Forward	Reverse
Pax7	CGATTAGCCGAGTGCTCAGAA	CCAGACGGTTCCCTTTGTCG
Myod1	CGCCACTCCGGGACATAG	GAAGTCGTCTGCTGTCTCAAAGG
Myf5	CAGCCCCACCTCCAACTG	GGGACCAGACAGGGCTGTTA
Myog	TCCAGTACATTGAGCGCCTAC	GCTGTGGGAGTTGCATTCAC
Osr1	CCTGTATGGTTTCAGCGCTC	TGGCTTAGGGTGAATGACGT
Gapdh	CTGCACCACCAACTGCTTAG	GGATGCAGGGATGATGTTCT
TGFβ1	GACCCCCACTGATACGCCTG	GCGCTGAATCGAAAGCCCTG
TGFβ2	CCGGAGGTGATTTCCATCTA	GCGGACGATTCTGAAGTAGG
TGFβ3	GATGAGCACATAGCCAAGCA	ATTGGGCTGAAAGGTGTGAC
CD68	GGCGGTGGAATACAATGTGTCC	AGCAGGTCAAGGTGAACAGCTG
CD86	ACGTATTGGAAGGAGATTACAGCT	TCTGTCAGCGTTACTATCCCGC
CD163	GGCTAGACGAAGTCATCTGCAC	CTTCGTTGGTCAGCCTCAGAGA
CD206	GTTCACCTGGAGTGATGGTTCTC	AGGACATGCCAGGGTCACCTTT
Arg1	AACACGGCAGTGGCTTTAACC	GGTTTTCATGTGGCGCATTC
Albumin	CTGCAATCCTGAACCGTGT	TTCCACCAGGGATCCACTAC

Supplementary Table 2 Primary antibodies

Antibody	Clone	Conjugate	Concentration / Dilution	Source
**Mouse anti-MHC3 (eMHC)**	Monoclonal	Unconjugated	1:50	DSHB
**Mouse anti-MF20 (MYH1)**	Monoclonal	Unconjugated	1:100	DSHB
**Mouse anti-Pax7 (supernatant)**	Monoclonal	Unconjugated	1:20	DSHB
**Guinea pig anti-Pax7**	Polyclonal	Unconjugated	1:100	C. Birchmeier
**Mouse anti-Ki67**	Monoclonal	B56	1:100	BD Biosciences
**Rabbit anti-Ki67**	Polyclonal	Unconjugated	1:1000	Abcam
**Rabbit anti-laminin**	Polyclonal	Unconjugated	5 µg ml-1	Sigma-Aldrich
**Goat anti-Collagen VI**	Polyclonal	Unconjugated	1:200	Southern Biotechnology Associates
**Mouse anti-MyoD**	5.8A	Unconjugated	1:100	BD Biosciences
**Mouse anti-fibronectin**	Polyclonal	Unconjugated	1:500	Merck
**Phalloidin**		591⁄608	1:250	Thermo Fischer Scientific
**Rabbit anti-tSmad2/3**	D7G7	Unconjugated	1:1000	Cell Signaling
**Rabbit anti-pSmad2**	Ser465/467	Unconjugated	1:1000	Cell Signaling
**Rabbit anti-pSmad3**	Ser423/425	Unconjugated	1:1000	Cell Signaling
**Rabbit anti-GAPDH**	14C10	Unconjugated	1:2000	Cell Signaling
**Mouse anti-myogenin**	sc-12732	Unconjugated	1:100	Santa Cruz
**Mouse anti-vinculin**	V9131	Unconjugated	1:4000	Merck
**Rabbit-anti-CD80**	Polyclonal	Unconjugated	1:150	Invitrogen
**Rat-anti-CD206**	MR5D3	Unconjugated	1:150	Bio-Rad
**Rabbit-anti-Perilipin A/B**	Polyclonal	Unconjugated	1:300	Sigma-Aldrich
**Goar-anti-PDGFRA**	Polyclonal	Unconjugated	1:200	R & D systems

Supplementary Table 3 Secondary antibodies

Antibody	Conjugate(s)	Source
Donkey anti-mouse	Alexa Fluor 488, 568 and 680	Invitrogen
Donkey anti-rabbit	Alexa Fluor 488, 568 and 681	Invitrogen
Donkey anti-goat	Alexa Fluor 488, 568 and 682	Invitrogen
Goat anti-guinea pig	Alexa Fluor 682	Invitrogen

Supplementary Table 4 FACS antibodies

Antibody	Clone	Conjugate	Concentration/dilution	Source
**Anti mouse CD31**	390	APC conjugated	3 µg ml^{-1}	Invitrogen
**Anti mouse CD45**	30-F11	APC conjugated	6 µg ml^{-1}	Invitrogen
**Anti mouse TER119**	TER-119	APC conjugated	6 µg ml^{-1}	Invitrogen
**Anti rat a7-integrin**	R2F2	PE conjugated	1.5 µg ml^{-1}	Ablab
**Anti mouse Ly-6A/E (Sca-1)**	D7	APC-Cy7 conjugated	1.5 µg ml^{-1}	Biolegend
**CD45**	30-F11	AF488	0.25 µg ml-1	BioLegend, San Diego, CA, USA
**CD3e**	145-2C11	PE-Cy7	0.4 µg ml-1	BioLegend, San Diego, CA, USA
**CD19**	6D5	PE-Cy7	0.2 µg ml-1	BioLegend, San Diego, CA, USA
**CD335**	29A1.4	PE-Cy7	0.6 µg ml-1	BioLegend, San Diego, CA, USA
**CD11c**	N418	PerCP-Cy5.5	0.4 µg ml-1	BioLegend, San Diego, CA, USA
**CD11b/Mac-1**	M1/70	BV510	0.4 µg ml-1	BioLegend, San Diego, CA, USA
**Ly6-G**	1A8	APC-Fire750	0.6 µg ml-1	BioLegend, San Diego, CA, USA
**MHC class II**	M5/114.15.2	BV785	0.05 µg ml-1	BioLegend, San Diego, CA, USA
**CD80**	16-10A1	BV650	0.2 µg ml-1	BioLegend, San Diego, CA, USA
**CD86**	GL-1	BV421	0.2 µg ml-1	BioLegend, San Diego, CA, USA
**CD163**	TNKUPJ	PE	0.1 µg ml-1	ThermoFisher, Waltham, MA, USA
**VEGF**	VG1	AF647	0.225 µg ml-1	Novus Biologicals, Littleton, CO, USA
**CD206**	C068C2	PE-Dazzle594	0.2 µg ml-1	BioLegend, San Diego, CA, USA
