# Supplementary material for: Odd skipped-related 1 controls the pro-regenerative response of fibro-adipogenic progenitors
Source: NPJ Regen Med. 2023 Apr 5;8:19. doi: 10.1038/s41536-023-00291-6 (PMC10076435; doi:10.1038/s41536-023-00291-6)
Supplement: Supplementary file 2 — Reporting Summary [file 41536_2023_291_MOESM2_ESM.pdf]

nature portfolio

Corresponding author(s): Sigmar Stricker

Last updated by author(s): Mar 3, 2023

Reporting Summary

Nature Portfolio wishes to improve the reproducibility of the work that we publish. This form provides structure for consistency and transparency in reporting. For further information on Nature Portfolio policies, see our Editorial Policies and the Editorial Policy Checklist.

Statistics

For all statistical analyses, confirm that the following items are present in the figure legend, table legend, main text, or Methods section.

n/a	Confirmed	
☐	☒	The exact sample size (*n*) for each experimental group/condition, given as a discrete number and unit of measurement
☐	☒	A statement on whether measurements were taken from distinct samples or whether the same sample was measured repeatedly
☐	☒	The statistical test(s) used AND whether they are one- or two-sided<br>*Only common tests should be described solely by name; describe more complex techniques in the Methods section.*
☐	☒	A description of all covariates tested
☐	☒	A description of any assumptions or corrections, such as tests of normality and adjustment for multiple comparisons
☐	☒	A full description of the statistical parameters including central tendency (e.g. means) or other basic estimates (e.g. regression coefficient) AND variation (e.g. standard deviation) or associated estimates of uncertainty (e.g. confidence intervals)
☒	☐	For null hypothesis testing, the test statistic (e.g. *F*, *t*, *r*) with confidence intervals, effect sizes, degrees of freedom and *P* value noted<br>*Give P values as exact values whenever suitable.*
☒	☐	For Bayesian analysis, information on the choice of priors and Markov chain Monte Carlo settings
☒	☐	For hierarchical and complex designs, identification of the appropriate level for tests and full reporting of outcomes
☒	☐	Estimates of effect sizes (e.g. Cohen's *d*, Pearson's *r*), indicating how they were calculated

Our web collection on statistics for biologists contains articles on many of the points above.

Software and code

Policy information about availability of computer code

Data collection	n/a
Data analysis	n/a

For manuscripts utilizing custom algorithms or software that are central to the research but not yet described in published literature, software must be made available to editors and reviewers. We strongly encourage code deposition in a community repository (e.g. GitHub). See the Nature Portfolio guidelines for submitting code & software for further information.

Data

Policy information about availability of data

All manuscripts must include a data availability statement. This statement should provide the following information, where applicable:

- Accession codes, unique identifiers, or web links for publicly available datasets
- A description of any restrictions on data availability
- For clinical datasets or third party data, please ensure that the statement adheres to our policy

All sequencing data are available via the BioProject accession number PRJNA938360.

Human research participants

Policy information about studies involving human research participants and Sex and Gender in Research.

Reporting on sex and gender	n/a
Population characteristics	n/a
Recruitment	n/a
Ethics oversight	n/a

Note that full information on the approval of the study protocol must also be provided in the manuscript.

Field-specific reporting

Please select the one below that is the best fit for your research. If you are not sure, read the appropriate sections before making your selection.

☒ Life sciences ☐ Behavioural & social sciences ☐ Ecological, evolutionary & environmental sciences

For a reference copy of the document with all sections, see nature.com/documents/nr-reporting-summary-flat.pdf

Life sciences study design

All studies must disclose on these points even when the disclosure is negative.

Sample size	No sample size calculation was performed, as there was no expected effect size. Sample numbers were used according to accepted standards in the field.
Data exclusions	No data were excluded.
Replication	All experiments have been replicated with independent biological samples as stated in the manuscript.
Randomization	not applicable; use of genetically modified inbred mouse strains.
Blinding	Non-blinded. For most analyses, automated quantification was performed.

Reporting for specific materials, systems and methods

We require information from authors about some types of materials, experimental systems and methods used in many studies. Here, indicate whether each material, system or method listed is relevant to your study. If you are not sure if a list item applies to your research, read the appropriate section before selecting a response.

Materials & experimental systems

n/a	Involved in the study	
☐	☒	Antibodies
☐	☒	Eukaryotic cell lines
☒	☐	Palaeontology and archaeology
☐	☒	Animals and other organisms
☒	☐	Clinical data
☒	☐	Dual use research of concern

Methods

n/a	Involved in the study	
☒	☐	ChIP-seq
☐	☒	Flow cytometry
☒	☐	MRI-based neuroimaging

Antibodies

Antibodies used	A list of antibodies can be found in the supplementary materials
Validation	Antibodies were validated by the manufacturer

Eukaryotic cell lines

Policy information about cell lines and Sex and Gender in Research

Cell line source(s)	C2C12 cells were derived from ATCC
Authentication	Myogenic differentiation assay
Mycoplasma contamination	Cell lines were regularly tested for mycoplasma contamination
Commonly misidentified lines (See ICLAC register)	n/a

Animals and other research organisms

Policy information about studies involving animals; ARRIVE guidelines recommended for reporting animal research, and Sex and Gender in Research

Laboratory animals	Mouse; C57Bl6 background; CAGG-CreERt strain (B6.Cg-Tg(CAG-cre/Esr1*)5Amc/J),
Wild animals	n/a
Reporting on sex	n/a
Field-collected samples	n/a
Ethics oversight	Animal experiments were approved by the "Landesamt für Gesundheit und Soziales" Berlin, Germany

Note that full information on the approval of the study protocol must also be provided in the manuscript.

Flow Cytometry

Plots

Confirm that:

☒ The axis labels state the marker and fluorochrome used (e.g. CD4-FITC).

☒ The axis scales are clearly visible. Include numbers along axes only for bottom left plot of group (a 'group' is an analysis of identical markers).

☒ All plots are contour plots with outliers or pseudocolor plots.

☒ A numerical value for number of cells or percentage (with statistics) is provided.

Methodology

Sample preparation	Isolation of cells from skeletal muscle by enzymatic tissue digestion.
Instrument	CytoFlex LX
Software	FlowJo software (Tree Star, Ashland, OR, USA)
Cell population abundance	N/A
Gating strategy	The gating strategy for FAP and MuSC isolation is shown in Supplementary Fig. 1b. The gating strategy for immune cell analysis was performed exactly as described in Bucher et al. (2022, PMID: 36028760). Figures illustrating the gating strategy can be found in supplementary information of this reference (Supplementary Figure 5-8). A corresponding statement is also included in the materials and methods section of the main manuscript.

☒ Tick this box to confirm that a figure exemplifying the gating strategy is provided in the Supplementary Information.
